# First fossil of an oestroid fly (Diptera: Calyptratae: Oestroidea) and the dating of oestroid divergences

**DOI:** 10.1371/journal.pone.0182101

**Published:** 2017-08-23

**Authors:** Pierfilippo Cerretti, John O. Stireman, Thomas Pape, James E. O’Hara, Marco A. T. Marinho, Knut Rognes, David A. Grimaldi

**Affiliations:** 1 Dipartimento di Biologia e Biotecnologie ‘Charles Darwin’, Sapienza Università di Roma, Rome, Italy; 2 Department of Biological Sciences, Wright State University, Dayton, OH, United States of America; 3 Natural History Museum of Denmark, University of Copenhagen, Copenhagen, Denmark; 4 Canadian National Collection of Insects, Agriculture and Agri-Food Canada, Ottawa, Ontario, Canada; 5 Laboratório de Morfologia e Evolução de Diptera, Departamento de Biologia, Faculdade de Filosofia, Ciências e Letras, Universidade de São Paulo, São Paulo, SP, Brazil; 6 Departamento de Ecologia, Zoologia e Genética, Instituto de Biologia, Universidade Federal de Pelotas, Pelotas, RS, Brazil; 7 University of Stavanger, Faculty of Arts and Education, Department of Early Childhood Education, Stavanger, Norway; 8 Division of Invertebrate Zoology, American Museum of Natural History, New York, United States of America; University of Michigan, UNITED STATES

## Abstract

Calyptrate flies include about 22,000 extant species currently classified into Hippoboscoidea (tsetse, louse, and bat flies), the muscoid grade (house flies and relatives) and the Oestroidea (blow flies, bot flies, flesh flies, and relatives). Calyptrates are abundant in nearly all terrestrial ecosystems, often playing key roles as decomposers, parasites, parasitoids, vectors of pathogens, and pollinators. For oestroids, the most diverse group within calyptrates, definitive fossils have been lacking. The first unambiguous fossil of Oestroidea is described based on a specimen discovered in amber from the Dominican Republic. The specimen was identified through digital dissection by CT scans, which provided morphological data for a cladistic analysis of its phylogenetic position among extant oestroids. The few known calyptrate fossils were used as calibration points for a molecular phylogeny (16S, 28S, CAD) to estimate the timing of major diversification events among the Oestroidea. Results indicate that: (a) the fossil belongs to the family Mesembrinellidae, and it is identified and described as *Mesembrinella caenozoica* sp. nov.; (b) the mesembrinellids form a sister clade to the Australian endemic *Ulurumyia macalpinei* (Ulurumyiidae) (McAlpine’s fly), which in turn is sister to all remaining oestroids; (c) the most recent common ancestor of extant Calyptratae lived just before the K–Pg boundary (ca. 70 mya); and (d) the radiation of oestroids began in the Eocene (ca. 50 mya), with the origin of the family Mesembrinellidae dated at ca. 40 mya. These results provide new insight into the timing and rate of oestroid diversification and highlight the rapid radiation of some of the most diverse and ecologically important families of flies. ZooBank accession number–urn:lsid:zoobank.org:pub:0DC5170B-1D16-407A-889E-56EED3FE3627.

## Introduction

The spectacular episodes of ‘explosive’ diversification that have occurred scattered in the history of life have always fascinated evolutionary biologists. Radiations are even more intriguing when the resulting species diversity and abundance we observe today contrasts with a fossil record that is sparse or even entirely lacking. In this respect, the calyptrate Diptera (e.g., tsetse, house flies, blow flies, and related groups) are a good example. Calyptrate flies comprise one of the major clades of Schizophora, the latter representing the most rampant radiation of Diptera ever [1] and one of the largest radiations of insects within the Cenozoic [2]. For oestroids—the most diverse group within the calyptrates—definitive fossils have been entirely lacking.

The approximately 22,000 extant species of calyptrates [3] (about 14% of all flies) are abundantly represented in nearly all terrestrial ecosystems, from tropical forests, savannas, and deserts to the extreme High Arctic, often playing key roles in the environment as decomposers, parasites, parasitoids, vectors of pathogens, and pollinators [4]. Calyptrates include some of the most common and well-known synanthropic scavengers on Earth such as the house fly (*Musca domestica* Linnaeus; Muscidae), the lesser house fly (*Fannia scalaris* (Fabricius); Fanniidae), and the blue bottle flies (*Calliphora* spp.; Calliphoridae); such notorious blood-feeders as the tsetse flies (*Glossina* spp.; Glossinidae) and the stable fly (*Stomoxys calcitrans* (Linnaeus); Muscidae); as well as such economically important mammal parasites as the New World screw-worm fly (*Cochliomyia hominivorax* (Coquerel); Calliphoridae) and the human bot fly (*Dermatobia hominis* (Linnaeus, Jr.); Oestridae).

Calyptrates are currently classified into two superfamilies and one ‘grade’, which together contain 15 families: the Hippoboscoidea, comprising the Glossinidae and Hippoboscidae (louse flies and bat flies); the muscoid grade of families (‘Muscoidea’; house flies and relatives), comprising the Fanniidae, Muscidae, Anthomyiidae, and Scathophagidae; and the Oestroidea, comprising the Calliphoridae (blow flies, probably non-monophyletic), Mesembrinellidae, Mystacinobiidae (New Zealand bat fly), Oestridae (bot flies), Rhiniidae (rhiniid flies), Rhinophoridae (woodlouse flies), Sarcophagidae (flesh flies), Tachinidae (parasitoid flies), and most recently the Ulurumyiidae (McAlpine’s fly [5]). Nearly all recent studies are in agreement that the Calyptratae are a monophyletic group, the emerging phylogenetic scheme being one with a basal Hippoboscoidea and a monophyletic Oestroidea nested within a paraphyletic muscoid grade [1, 6, 7, 8, 9, 10] (but see Ding *et al*. [11] for an alternate hypothesis). Relationships within Oestroidea are complicated and not well resolved, with little agreement between morphology- and molecular-based studies [7, 12, 13, 14, 15, 16, 17, 18, 19].

The most recent phylogenetic analysis employing a relaxed molecular clock model suggests a calyptrate origin near the K–Pg boundary, 66 million years ago (mya) [1], but there are few reliable calyptrate fossils known so far: one stem-group anthomyiid (*Protanthomyia minuta* Michelsen) from Eocene Baltic amber (ca. 42 mya), which represents the oldest calyptrate known so far [20]; a few tsetse flies in shales from the latest Eocene of North America and the Oligocene of Europe (40–35 mya) [2]; and a few crown-group representatives of Anthomyiidae [21], Muscidae [22], and Hippoboscidae [23] from Dominican amber, which are imprecisely dated within the Miocene at 20–15 my [24].

Putative oestroid fossils include five mineralized puparia in a piece of ironstone from the Late Cretaceous (70 mya) of Canada, described as *Cretaformia fowleri* McAlpine and originally attributed to the Calliphoridae *sensu lato* [25]. This fossil was later treated as an unplaced schizophoran [2, 26], but even this placement may be questionable because neither mouthparts nor posterior spiracles are recognizable and only the annulation of the wrinkled surface bears some resemblance to a cyclorrhaphan puparium. Tertiary fossils that have been tentatively assigned to oestroid families are listed in Table 1. These are mostly compression fossils of larvae or adults that are currently assigned to Oestridae, Sarcophagidae, and Tachinidae [27]. However, these placements are best treated as speculative because no diagnostic characters conclusively support them [28, 29, 30]. It is worth noting that larvae of both Oestridae and Tachinidae are obligate endophages of living mammals and insects, respectively, with the free-living larval stage restricted to the short period when the mature larva leaves the host to pupate. Fossilisation of such larvae, which are free-living for such a short time, must be an extremely rare event. This improbability, along with the lack of diagnostic features, suggests that pre-Quaternary fossil larvae tentatively assigned to parasitic Oestroidea are misidentified.

**Table 1:** Putative oestroid fossils of the Tertiary.

		Eocene (56–34 mya)	Oligocene (34–23 mya)	Miocene (23–5 mya)	Undetermined Tertiary
**Oestridae**	*Adipterites obovatus* (Heer, 1864) (Lr) (Co)			Upper Freshwater-Molasse Formation, Sarmatian (12.7–11.6 mya), Germany 47.7°N, 8.9°E	
**Oestridae**	*Agiebelia ignota* (Townsend, 1921) (Lr) (Co)				locality undetermined
**Oestridae**	*Cuterebra ascarides* (Scudder, 1877) (Lr) (Co)	Green River Formation, Bridgerian (50.3–46.2 mya), Colorado, USA 39.0°N, 108.0°W			
**Oestridae**	*Cuterebra bibosa* (Scudder, 1877) (Lr) (Co)	Green River Formation, Bridgerian (50.3–46.2 mya), Colorado, USA 39.0°N, 108.0°W			
**Oestridae**	*Dermatobia hydropica* (Scudder, 1877) (Lr) (Co)	Green River Formation, Bridgerian (50.3–46.2 mya), Colorado, USA 39.0°N, 108.0°W			
**Oestridae**	*Novoberendtia baltica* (Townsend, 1921) (Lr) (Am)	Baltic amber (?) (no further information available)			
**Tachinidae**	*Lithexorista scudderi* Townsend, 1921 (Ad) (Co)	Green River Formation, Bridgerian (50.3–46.2 mya), Wyoming, USA 41.6°N, 109.6°W			
**Tachinidae**	*Lithotachina antiqua* (Heer, 1849) (Ad) (Co)			Upper Freshwater-Molasse Formation, Sarmatian (12.7–11.6 mya), Germany 47.7°N, 8.9°E	
**Tachinidae**	*Muscidites deperditus* Heyden & Heyden, 1866 (Lr) (Co)		Chattian		
			(28.4–23.0 mya), Germany 49.7°N, 8.2°E		
**Tachinidae**	*Tachina* sp. (?) (Co)			Switzerland [no further information available]	
**Tachinidae**	*Vinculomusca vinculata* (Scudder, 1877) (Lr) (Co)	Green River Formation, Bridgerian (50.3–46.2 mya), Colorado, USA 39.0°N, 108.0°W			
**Tachinidae**	Undetermined Tachinidae (Eg) (Am)		Dominican mines (15–20 mya), Dominican Republic		
**Sarcophagidae**	*Sarcophaga* sp. (?Lr) (Am)	Baltic amber (?) (no further information available)			

Abbreviations: Ad = adult, Am = amber, Co = compression, Eg = egg, Lr = larva. Data from Evenhuis [27], the Fossilworks website (http://fossilworks.org/bridge.pl?a=home (accessed July 7, 2016)) and from original descriptions.

The putative plano-convex dipteran egg attached to the pronotum of a leaf beetle (Chrysomelidae) preserved in Dominican amber [31, 32] is particularly interesting. Within Diptera, the plano-convex egg shape has evolved independently in at least five lineages of arthropod parasitoids: once in the Rhinophoridae, three times in the Tachinidae (Eutherini, Exoristinae, and Phasiinae), and once in the Muscidae (*Eginia* Robineau-Desvoidy) [33, 34, 35, 36]. However, while the size, shape, and position of the object on the beetle’s body are suggestive of a tachinid egg, there are no other morphological clues to support or reject such a conclusion.

There are a few Quaternary (sub)fossils and copal inclusions of Oestroidea [27], but they are not discussed here because they have little bearing on oestroid evolution. Two genera in this category, *Paleotachina* Townsend and *Electrotachina* Townsend, were long thought to be Eocene fossils of Tachinidae (and were catalogued as such [27]) but were revealed as copal inclusions [37] and later assigned to the Muscidae and Sarcophagidae, respectively [30]. Not included in Table 1 are brief notes on Tachinidae and Calliphoridae in Dominican amber [31, 38] or the record (with photograph) of a specimen of Calliphoridae (possibly *Chrysomya* Robineau-Desvoidy) from ‘a piece of nearly colourless amber’ [39]. The material upon which these reports were based has not been further described or illustrated and cannot be evaluated until more detailed information is published.

The absence of unambiguous pre-Quaternary oestroid fossils necessitated the dating of oestroid lineages from tectonic or other geophysical events considered causative for patterns in current distributions, like the closure of the Transantarctic corridor (minimum age for dispersal of nasal bot flies into Australia [29]), the collision of the African and Eurasian plates (maximum age for diversification of the rhino and horse stomach bot flies [29]), and the submersion of the Isthmus of Panama and the existence of a Protoantillean corridor (vicariance dating for some flesh fly lineages [40]). Nevertheless, this approach is of limited use in dating clades [41] because it assumes that the distributions of organisms at the time of these events were static and constrained by geophysical events, with little consideration for dispersal events and the widespread ebb and flow in distributions, which fossil data often indicates actually happened in most animal and plant taxa (probably in response to palaeoclimatic change) [42].

This paper describes the first unambiguous fossil of Oestroidea based on a perfectly preserved male fly discovered in amber from the Dominican Republic. We identify and characterize the specimen through digital dissection of its terminalia by CT scans, which provide morphological data for a cladistic analysis of the phylogenetic position of the fossil among extant oestroid lineages. We use the few known calyptrate fossils as calibration points for a preliminary molecular phylogeny (using 16S, 28S and CAD) to estimate the timing of major diversification events among the Oestroidea.

## Results

### Systematics

Order Diptera

Superfamily Oestroidea

Family Mesembrinellidae

Genus *Mesembrinella* Giglio-Tos, 1893.

*Mesembrinella caenozoica* sp. nov.

Figs 1 and 2

**Fig 1:**
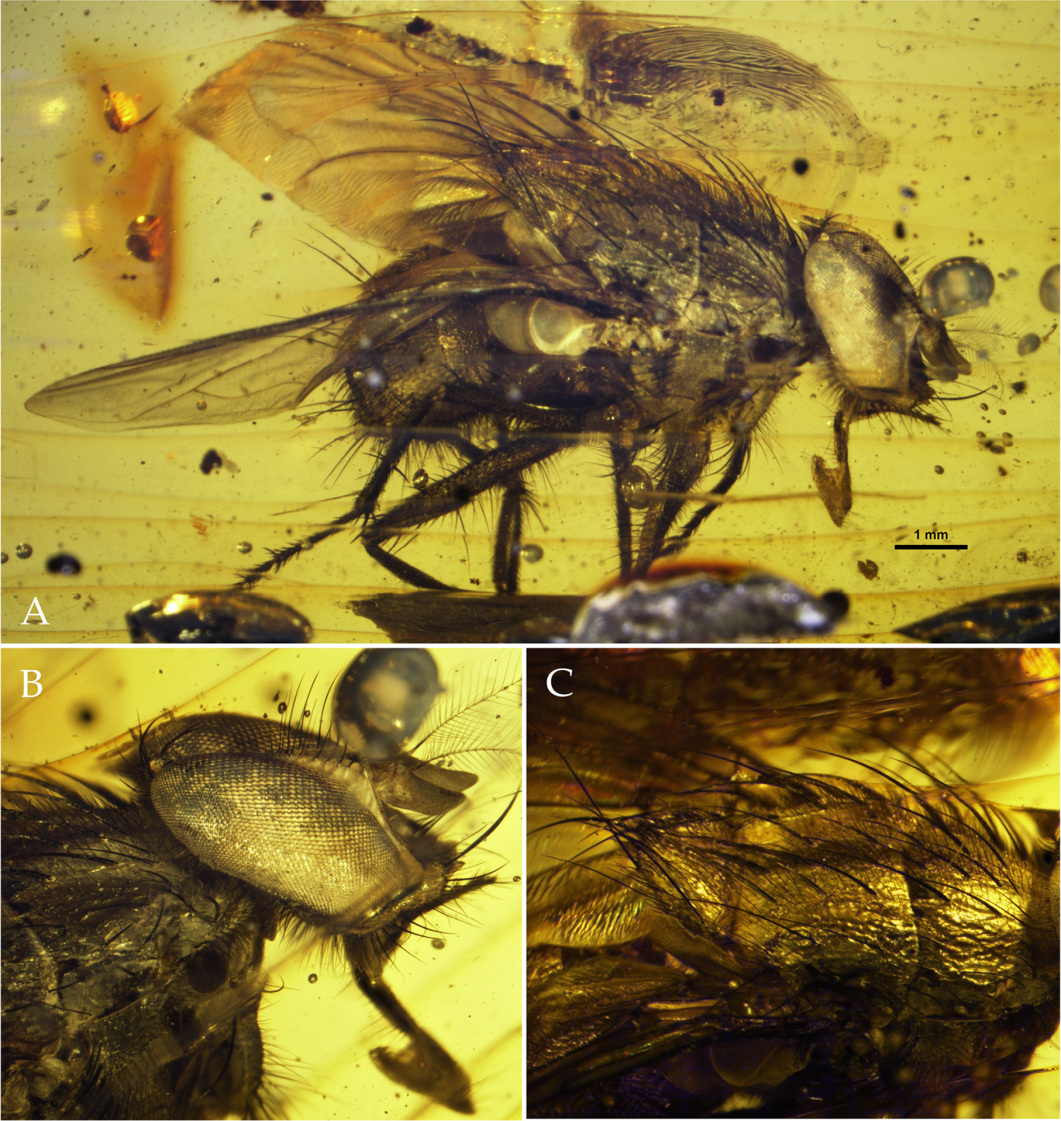
Holotype of *Mesembrinella caenozoica* sp. nov. (A) habitus in right dorsolateral view. (B) head and part of thorax in right dorsolateral view. (C) thorax in right dorsolateral view.

**Fig 2:**
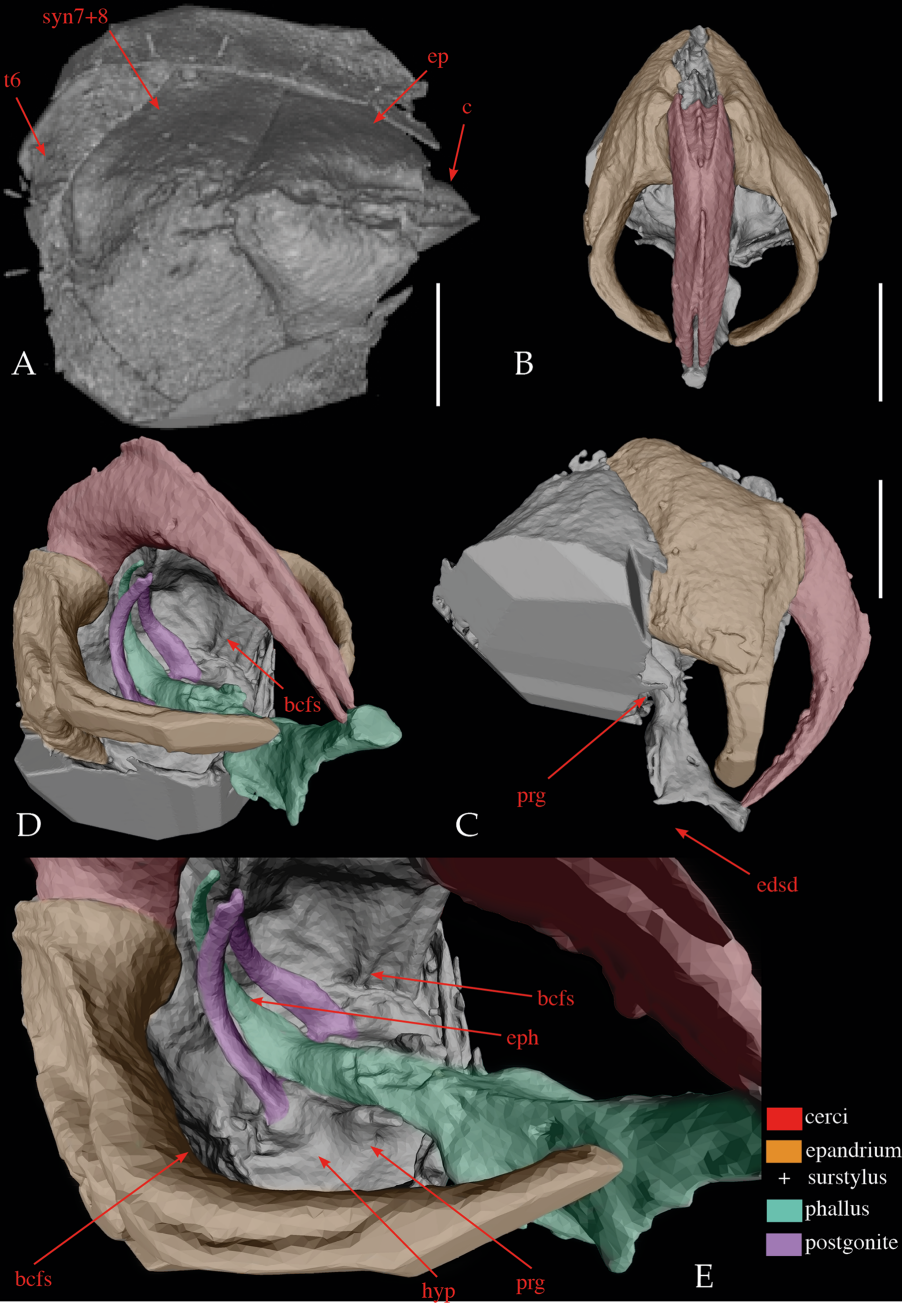
Holotype of *Mesembrinella caenozoica* sp. nov., CT scan of the terminalia. (A) tergite 6, sytergosternite 7+8, epandrium and cerci in posterior view. (B) epandrial complex in posterior view. (C) epandrial complex and phallus in left lateral view. (D) terminalia in left ventrolateral view. (E) detail of phallus and hypandrial complex in left ventrolateral view. Abbreviations: bcfs = bacilliform sclerite; c = cerci; edsd = tip of extension of dorsal sclerite of distiphallus; ep = epandrium; eph = epiphallus; hyp = hypandrium; prg = pregonite; syn7+8 = syntergosternite 7+8; t6 = tergite 6. Scale bars: 0.4 mm.

Type material. Holotype male, a Dominican amber inclusion of Miocene age, housed in the American Museum of Natural History. Additional details are given under Materials and Methods.

Etymology. The specific epithet ‘*caenozoica*’ alludes to the name of the Cenozoic Era (from Greek *kainos*, meaning ‘new’, and *zoe*, meaning ‘life’), which covers the period from ca. 66 mya to the present day. The epithet should be treated as a Latin adjective.

Diagnosis. A medium-sized fly (body length: ca. 8.5 mm) (Fig 1A), readily distinguishable from extant mesembrinellids by the following combination of character states: prementum about 0.65–0.70 times as long as height of head; labella broad and about 4/5 as long as prementum; palpus sub-cylindrical, about as long as antenna (Fig 1A and 1B); proepisternal depression with pale, hair-like setulae; postpronotum with three setae arranged in a triangle; scutum with 3(presutural) + 3(postsutural) dorsocentral setae (Fig 1C); 3 katepisternal setae; metathoracic spiracular lappet without setae; general setulae of postpronotum, scutum and scutellum relatively long and suberect; posterolateral margin of lower calypter with long trichia; stem vein bare; vein R_4+5_ with fine setulae from base to about 2/3 of distance to crossvein r-m; abdominal tergite 3 with two long, erect median marginal setae and two lateral marginal setae (Fig 1A); setae on abdominal sternites normally developed and not arranged in two rows; abdominal tergite 6 broad, not indented posteriorly and not fused to syntergosternite 7+8 (Fig 2A); cerci long, narrow and evenly curved anteroventrally (Fig 2B–2D); lateroventral lobes of distiphallus well developed (Fig 2D and 2E); scale-like vestiture of lateroventral lobes of distiphallus not visible; narrow lateral projection of lateroventral lobes of distiphallus present; acrophallus well developed, sub-cylindrical (Fig 2C and 2D).

Description: S1 Text.

### Morphological cladistic analysis

**Fig 3:**
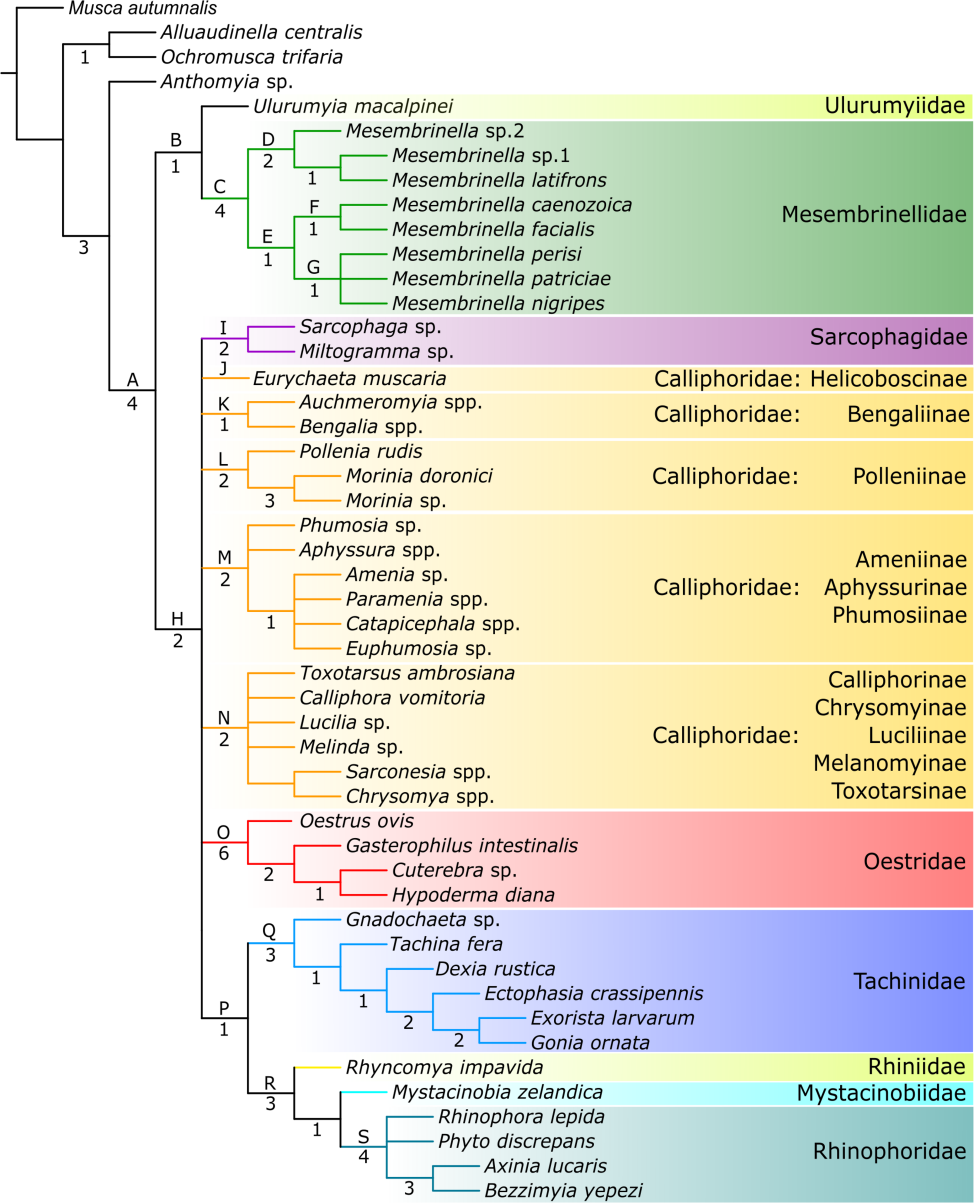
Strict consensus cladogram of major lineages of Oestroidea from 30 most parsimonious trees under equal weights (length = 258 steps; C.I. = 0.400; R.I. = 0.754) generated in TNT 1.5, from analysis of the morphological dataset (S1 Dataset). Capital letters above branches indicate branches as discussed in the text. Numbers below branches indicate Bremer supports values (BS).

The heuristic tree searches using parsimony with equal weights yielded 30 most parsimonious trees (MPTs) (tree length: 258 steps; Consistency Index: 0.400; Retention Index: 0.754). All of these MPTs recovered the superfamily Oestroidea (clade A) and the family Mesembrinellidae (clade C) as monophyletic, both with a Bremer support value (BS) of 4 (strict consensus tree in Fig 3). Optimization of the character transformations performed on the favoured MPT (S1 Fig) revealed that mesembrinellid monophyly relies on one nonhomoplastic (56:1, spermathecae very elongated), and eight homoplastic apomorphies (8:1, prothoracic spiracle with drop-shaped dorsal opening; 10:1, postalar wall setose; 11:1, metasternal area setose; 12:1, coxopleural streak absent; 19:1, metathoracic spiracle large; 24:0, excavation of syntergite 1+2 extending to posterior margin; 31:1, bend of M characteristically broadly rounded; 33:1, hind surface of hind coxa setose). The first split divides the family Mesembrinellidae into two subclades: clade D including species formerly ascribed to subfamily Mesembrinellinae *sensu* Guimarães [43] (Table 2), and clade E including species formerly assigned to subfamilies Laneellinae (*M*. *nigripes* (Guimarães) and *M*. *perisi* (Mariluis)), Souzalopesiellinae (*M*. *facialis* (Aldrich)) (Table 2), plus *M*. *patriciae* Wolff and *M*. *caenozoica* sp. nov. The position of *M*. *caenozoica* as sister to *M*. *facialis* (clade F) is supported by one nonhomoplastic apomorphy (45:1, lateral projection of lateroventral lobe of distiphallus present) (see S1 Dataset).

**Table 2:** Valid names for all extant species and subspecies of Mesembrinellidae.

	‘LUMPERS’		‘SPLITTERS’	
***Mesembrinella* clade**		Laneellinae	Souzalopesiellinae	Mesembrinellinae
	***M*. *abaca*** (Hall)			***Mesembrinella***
	***M*. *aeneiventris*** (Wiedemann)			***Huascaromusca***
	***M*. *andina*** (Wolff, Bonatto & Carvalho)			***Thompsoniella***
	***M*. *anomala*** (Guimarães)			***Thompsoniella***
	***M*. *apollinaris*** Séguy			***Mesembrinella***
	***M*. *batesi*** Aldrich			***Mesembrinella***
	***M*. *bellardiana bellardiana*** Aldrich			***Mesembrinella***
	***M*. *bellardiana fuscicosta*** Séguy			***Mesembrinella***
	***M*. *benoisti*** (Séguy)			***Eumesembrinella***
	***M*. *bequaerti*** (Séguy)			***Huascaromusca***
	***M*. *bicolor*** (Fabricius)			***Mesembrinella***
	***M*. *bolivar*** (Bonatto)			***Giovanella***
	***M*. *brunnipes*** Surcouf			***Mesembrinella***
	***M*. *carvalhoi*** (Wolff et al.)			***Huascaromusca***
	***M*. *cordillera*** (Wolff & Ramos-Pastrana)			***Huascaromusca***
	***M*. *currani*** Guimarães			***Mesembrinella***
	***M*. *cyaneicincta cyaneicincta*** (Surcouf)			***Eumesembrinella***
	***M*. *cyaneicincta pauciseta*** Aldrich			***Eumesembrinella***
	***M*. *decrepita*** (Séguy)			***Huascaromusca***
	***M*. *flavicrura*** Aldrich			***Mesembrinella***
	***M*. *lara*** (Bonatto)			***Huascaromusca***
	***M*. *latifrons*** Mello			***Albuquerquea***
	***M*. *obscura*** (Wolff)			***Huascaromusca***
	***M*. *patriciae*** Wolff			***Mesembrinella***
	***M*. *peregrina*** Aldrich			***Mesembrinella***
	***M*. *pictipennis*** Aldrich			***Mesembrinella***
	***M*. *purpurata*** (Aldrich)			***Huascaromusca***
	***M*. *quadrilineata*** (Fabricius)			***Eumesembrinella***
	***M*. *randa*** (Walker)			***Eumesembrinella***
	***M*. *semiflava*** (Aldrich)			***Huascaromusca***
	***M*. *semihyalina*** Mello			***Mesembrinella***
	***M*. *spicata*** (Aldrich)			***Henriquella***
	***M*. *townsendi*** (Guimarães)			***Mesembrinella***
	***M*. *umbrosa*** Aldrich			***Mesembrinella***
	***M*. *uniseta*** Aldrich			***Huascaromusca***
	***M*. *volgelsangi*** (Mello)			***Huascaromusca***
	***M*. *xanthorrhina*** (Bigot)			***Mesembrinella***
***Laneella* clade**				
	***M*. *nigripes*** (Guimarães)	***Laneella***		
	***M*. *patriciae*** Wolff			***Mesembrinella***
	***M*. *perisi*** (Mariluis)	***Laneella***		
	***M*. *facialis*** (Aldrich)		***Souzalopesiella***	

Comparison between classification schemes of the family Mesembrinellidae: ‘lumpers’ (grey column) *vs*. ‘splitters’ (white columns).

### Molecular phylogenetic analyses and divergence time estimation

**Fig 4:**
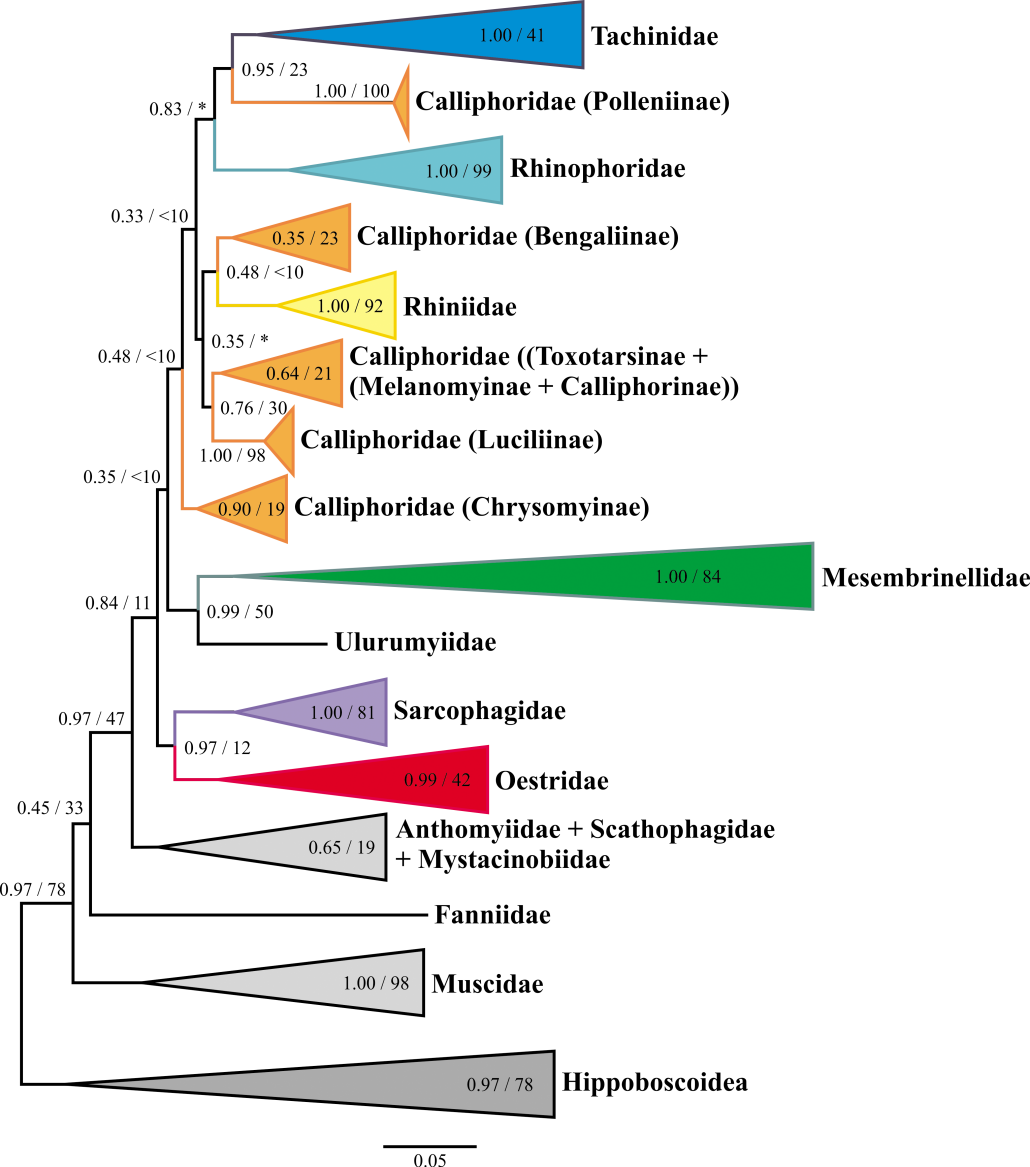
Bayesian phylogenetic reconstruction collapsed into clades at family and subfamily level of major lineages of Oestroidea from analyses of the combined (16S, 28S, CAD) dataset, generated in MrBayes (above branches left = Bayesian posterior probabilities; above branches right = maximum likelihood bootstrap support).

**Fig 5:**
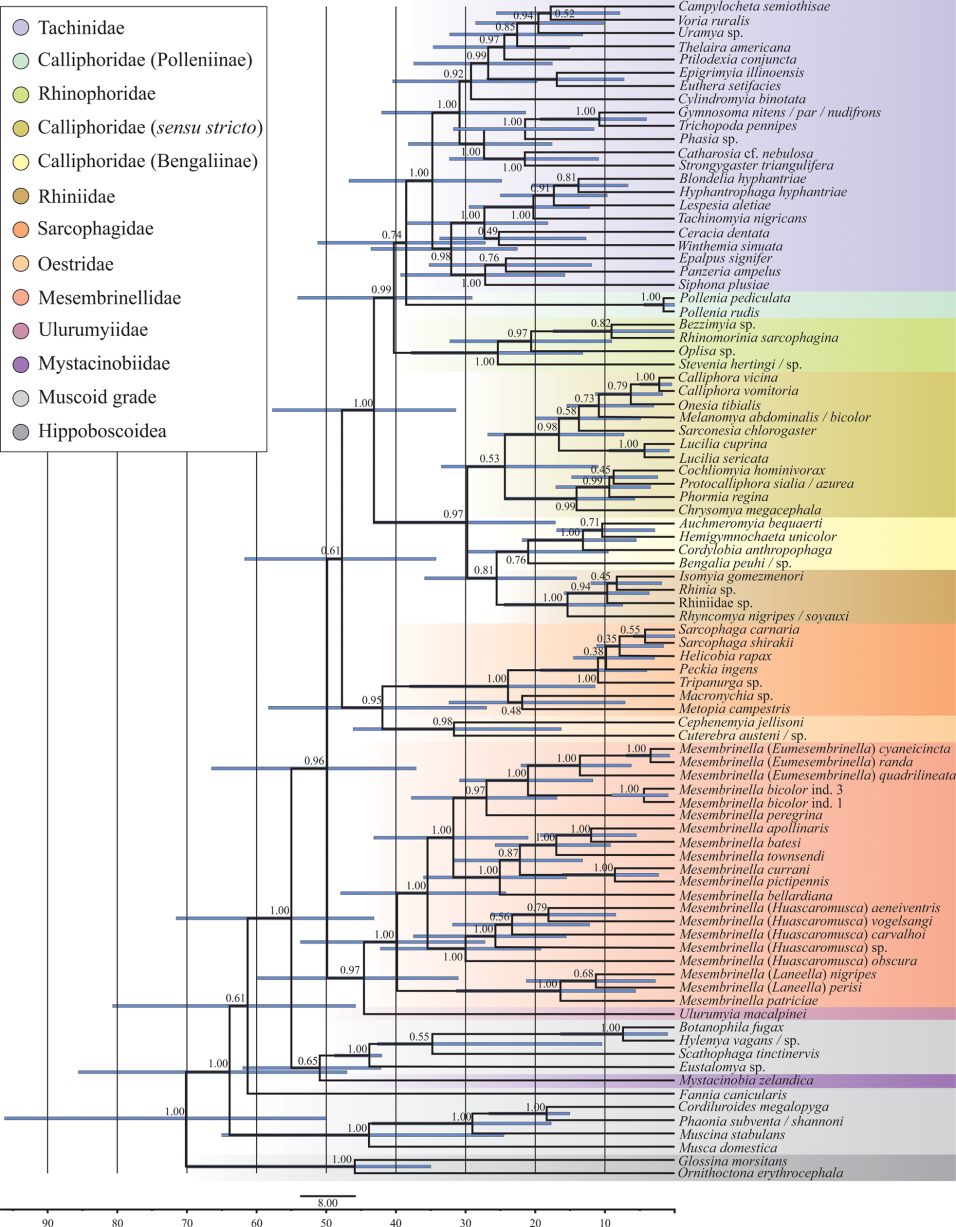
Bayesian inferred time-calibrated phylogeny of major lineages of Oestroidea from analysis of the combined (16S, 28S, CAD) dataset, generated in BEAST v. 2.4.0. The length of the light blue bars at nodes indicates 95% confidence intervals of node ages. Numbers indicate posterior probabilities. Time scale unit: 10 million years.

Overall, trees inferred via ML and Bayesian inference methods were similar in their reconstruction of clades (i.e., families/subfamilies), with relationships among major clades receiving, in general, low (< 50%) bootstrap support (bs; ML) and moderate (< 90%) posterior probabilities (pp; Bayesian) (Figs 4 and 5, S2 Fig, S3 Fig).

Despite partial reliance on the notoriously inconclusive 28S ribosomal gene [17], our analyses reconstructed relationships largely consistent with those of previous studies employing considerably more data, but fewer taxa (e.g., [16, 17]). For example, overall relationships among families are largely consistent with Wiegmann *et al*.’s [1] phylogeny of the order Diptera, with a paraphyletic muscoid grade, Oestridae as sister to Sarcophagidae, and Tachinidae sister to a clade of Calliphoridae, with Rhinophoridae somewhat removed from this latter clade. On the other hand, relationships within Oestroidea depart to some extent from those of Kutty *et al*. [7] and Marinho *et al*. [15] (e.g., position of Mesembrinellidae and Tachinidae), and as in these previous studies, relationships among major oestroid clades (families and calliphorid subfamilies) are not always well resolved.

Major inferred relationships are summarized below:

1. Calyptratae: In ML and Bayesian trees (Figs 4 and 5, S2 Fig, S3 Fig), Hippoboscoidea are sister to the remaining taxa with substantial support (78% bs; 0.97 pp; constrained in the BEAST analysis), with Muscidae, Fanniidae, and Anthomyiidae + Scathophagidae (with or without Mystacinobiidae) forming a paraphyletic grade of muscoid families from within which the Oestroidea arose. Node age estimates have broad confidence intervals (Fig 5), especially for deeper nodes. The median estimated age of the Calyptratae (basal node) is 67.5 my (95% CI: 49.8–96.2 mya).
2. Oestroidea: Oestroid ‘backbone’ relationships are supported by generally high posterior probabilities in Bayesian analyses, but are weakly resolved in the ML analyses (Fig 4, S2 Fig). The superfamily itself is well-supported in Bayesian analyses, with the exception of the Mystacinobiidae, which in some analyses emerge as sister to Anthomyiidae + Scathophagidae. The family Oestridae always emerges as sister taxon to the Sarcophagidae, with this clade being either sister to all remaining oestroids (MrBayes; Fig 4, S3 Fig) or sister to all oestroids except Mesembrinellidae + Ulurumyiidae (BEAST; Fig 5). The age of Oestroidea (minus *Mystacinobia*) is estimated to be 48.2 my (37.1–66.5), that of Sarcophagidae + Oestridae is 40.7 my (27.0–58.3), and Sarcophagidae are estimated to be at least 23 million years old (myo) (11.4–38.0).
3. Tachinidae: Relationships among tachinid taxa largely mirror those recovered from recent analyses using much larger data sets [17]. Tachinid monophyly is supported (41% bs, 1.0 pp) and all analyses reconstruct the Polleniinae (Calliphoridae) as sister to Tachinidae (as in Winkler *et al*. [17]). Bayesian analyses (BEAST and MrBayes) support Rhinophoridae as sister to Tachinidae + Polleninae (0.99 and 0.83 pp respectively) (Figs 4 and 5, S3 Fig), a relationship also recovered in the best ML tree (S2 Fig). The estimated age of Tachinidae is remarkably young for such a diverse family, at only 33.7 my (24.8–46.7).
4. Calliphoridae *sensu lato*: Relationships among the various clades of Calliphoridae *s*.*l*. vary among analyses, with the Bayesian trees indicating either a monophyletic clade of calliphorids minus Mesembrinellidae and Polleniinae (BEAST, 0.97 pp, Fig 5), or a grade of two major lineages, 1) Chrysomyinae and 2) all remaining calliphorids (MrBayes and ML analyses), which are paraphyletic with regard to the clade Rhinophoridae + (Tachinidae + Polleniinae): (Fig 4, S2 Fig, S3 Fig).
5. Mesembrinellidae: All analyses reconstruct Mesembrinellidae and Ulurumyiidae as sister taxa with medium to strong support (50% bs, 0.97–0.99 pp; Figs 4 and 5) (see also the morphology-based reconstruction in Fig 3). Relationships within Mesembrinellidae generally correspond to those obtained by Marinho *et al*. [19], which is not surprising given that analyses are based largely on the same data. *Mesembrinella nigripes*, *M*. *perisi* and *M*. *patriciae* form a clade (*Laneella* clade) sister to the remaining mesembrinellids (*Mesembrinella* clade). The age of the clade Mesembrinellidae + Ulurumyiidae is estimated to be 43.1 my (31.1–60.0), and the Mesembrinellidae themselves are estimated to be 38.8 myo (27.2–53.7).

## Discussion

### Morphological phylogeny of Oestroidea

Overall, the backbone relationships of oestroid families are poorly supported in our analysis. Although most families and subfamilies represented by at least two terminal taxa were reconstructed as monophyletic, the 30 most parsimonious trees vary widely in the reconstructed relationships among them (S1 Dataset). This ambiguity is apparent in the strict consensus cladogram of Fig 3, where a monophyletic Mesembrinellidae + Ulurumyiidae (clade B; BS: 1) is sister to clade H (BS: 2), which includes all the remaining oestroid taxa as part of a large basal polytomy. Within clade H, families Sarcophagidae (clade I, BS: 2), Oestridae (clade O, BS: 6), Tachinidae (clade Q, BS: 3), and Rhinophoridae (clade S, BS: 4) are monophyletic (the Rhiniidae were represented by only one species and the Mystacinobiidae are monotypic), whereas the ‘Calliphoridae’ break into five clades as follows:

Clade J–Helicoboscinae (*Eurychaeta muscaria* (Meigen));

Clade K–Bengaliinae (*Bengalia* Robineau-Desvoidy, *Auchmeromyia* Brauer & Bergenstamm) (BS: 1);

Clade L–Polleniinae (*Pollenia* Robineau-Desvoidy, *Morinia* Robineau-Desvoidy) (BS: 2);

Clade M–Ameniinae (*Amenia* Robineau-Desvoidy, *Catapicephala* Macquart, *Paramenia* Brauer & Bergenstamm) + Aphyssurinae (*Aphyssura* Hardy) + Phumosiinae (*Euphumosia* Malloch, *Phumosia* Robineau-Desvoidy) (BS: 2);

Clade N–Chrysomyinae (*Chrysomya*) + Toxotarsinae (*Toxotarsus*, *Sarconesia*) + Luciliinae (*Lucilia*) + Calliphorinae (*Calliphora*) + Melanomyinae (*Melinda*) (BS: 2).

Notably, clade P reconstructs the Tachinidae (clade Q) sister to a clade composed by Rhiniidae + (Mystacinobiidae + Rhinophoridae) (clade R; BS: 3).

### Mesembrinellid diversity and systematics, and the phylogenetic position of *Mesembrinella caenozoica*

Mesembrinellids are a small group of 38 extant species, traditionally classified into three subfamilies and nine genera [19, 43, 44] (Table 2, right column). However, this morphology-based generic and suprageneric classification, which is adopted by several authors, is largely unsupported by a hierarchical array of synapomorphies [45, 46, 47]. Moreover, recent and ongoing studies based on molecular data [19] do not corroborate monophyly of most of the non-monotypic nominal genera of this family. Accepting genera for which explicit evidence of monophyly is lacking, inevitably leads to taxonomic and nomenclatural instability. Coupling the recent results of phylogenetic analyses of the Mesembrinellidae with such a weak generic classification would trigger the redundant reassignment of several species to different genera, or the need to erect new monotypic genera to hold ‘non-fitting’ species, leading to a proliferation of names and confusion. For these reasons, we have chosen to adopt a more conservative classification scheme, as recently proposed by Vargas & Wood [48] and Moll [47], by lumping all the 38 valid species of mesembrinellids under the nominal genus *Mesembrinella*.

In the parsimony-based phylogenetic analysis the Dominican species is reconstructed within the Mesembrinellidae as sister to *Mesembrinella facialis*. Clade E (*M*. *facialis* + *M*. *caenozoica*) + (*M*. *patriciae* + *M*. *nigripes* + *M*. *perisi*) includes all the representatives of the former subfamilies Souzalopesiellinae and Laneellinae *sensu* Guimarães [43] plus *M*. *patriciae* (Table 2), which has been recently retrieved as sister to clade *M*. *nigripes* + *M*. *perisi* also on molecular grounds [19]. Clade E is sister to the other three mesembrinellid taxa analyzed, which were formerly classified in the nominal subfamily Mesembrinellinae *sensu* Guimarães [43] (Table 2). These results are in contrast to the morphological phylogenies of Toma & Carvalho [45] and Bonatto [46], in which a monophyletic *M*. *perisi* + *M*. *nigripes* (as *Laneella*) is the basal lineage of the Mesembrinellidae, and *M*. *facialis* (as *Souzalopesiella*) is sister to all mesembrinellids except the clade *M*. *perisi* + *M*. *nigripes*, but the results largely agree with the recent and more comprehensive study of Moll [47]. The molecular phylogeny of Marinho *et al*. [19] also reconstructed *M*. *perisi* + *M*. *nigripes* + *M*. *patriciae* as monophyletic and sister to the remaining Mesembrinellidae.

### Mesembrinellidae—Distribution and natural history

All extant mesembrinellid species are restricted in distribution to the rainforests of the Neotropical Region, from southern Mexico (Yucatan) to northern Argentina (Buenos Aires), with no records from the Caribbean islands except for Trinidad and Tobago [43]. The ecology of these flies is poorly known, but adults are shade-loving and occur almost exclusively within forests with a closed canopy. Occasional observations of mesembrinellids in clearings have been made during cloudy or rainy days. They are silent and fast fliers—although females fly more like big muscids, i.e., not as swift and agile as other large oestroids—mostly attracted to fermenting fruit, decaying animal matter and bird faeces [43]. Females of all species display pseudo-placental macrolarvipary, depositing one, relatively large, late first instar larva at a time, which has been nourished by secretions apparently produced by the spermathecae [49]. The few attempts of *in vitro* rearing of a selection of species on both animal and plant material have mostly failed (TP, unpubl.), except for *Mesembrinella nigripes*, which was successfully reared from first instar to adult on a variety of animal substrates, including dead snails, which were suggested as a possible primary resource for this species by Guimarães [43]. In fact, the successful breeding of *M*. *nigripes* on a diversity of decaying animal matter does not necessarily reflect its true breeding habits in the wild, but rather hints at a possible ancestral plasticity in this genus. This would be consistent with our phylogenetic reconstruction, according to which the mesembrinellids are sister to a macrolarviparous coprophage, the endemic eastern Australian *Ulurumyia macalpinei* Michelsen & Pape (see also the molecular phylogeny section).

According to Guimarães [43], mesembrinellids have two types of first instars that likely reflect different developmental strategies. The first instar of *M*. *nigripes*, *M*. *facialis* and *M*. *patriciae* is sub-conical or cylindrical in shape [19, 43] and has slender and pointed mouthhooks, whereas first instars of other mesembrinellids (e.g., *M*. *abaca* (Hall), *M*. *aeneiventris* (Wiedemann), *M*. *batesi* Aldrich, *M*. *bellardiana* Aldrich, *M*. *benoisti* (Séguy), *M*. *cyaneicincta* (Surcouf), *M*. *latifrons* Mello, *M*. *peregrina* Aldrich, *M*. *purpurata* (Aldrich), *M*. *quadrilineata* (Fabricius), *M*. *randa* (Walker) and *M*. *semihyalina* Mello) are ovoid in shape and the mouthhooks are dorsoventrally flattened and apically rounded (‘spatula-like’ of Guimarães [43]), a shape that is unusual among oestroid flies and would seem to indicate a special diet. Character states of larval *M*. *nigripes*, *M*. *facialis* and *M*. *patriciae* are possibly plesiomorphic with respect to those of the remaining mesembrinellids [43] (but see also Toma & Carvalho [45]).

Interestingly, the two other oestroid lineages that, according to our parsimony-based phylogenetic reconstruction, independently evolved a macrolarviparous reproductive strategy (Helicoboscinae and Ameniinae + relatives) attack dying or live land snails respectively [50, 51, 52, 53].

### *Mesembrinella caenozoica*–palaeoenvironment and extinction

Fossils can reveal much about the palaeoclimatic and palaeoenvironmental conditions that prevailed at the time when the organisms lived, and can sometimes provide indirect information about the co-occurrence of species that are lacking from the fossil record. For example, the discovery of specialized fossil pollinators and phytophages indicates the presence of their pollinizers or host plants respectively, in the same way as a diversity of fossil bloodsucking arthropods suggests a diversity of vertebrate hosts, or fossils of insects with aquatic life stages suggest the proximity of freshwater [54]. Paleo-environmental reconstructions [2, 31, 54] based on plant and animal inclusions of the Miocene Dominican amber deposits are of a diverse tropical rain forest ecosystem with clearings, ponds and streams of the sort that occurs there today. Coupling such reconstructions with the little we know about the ecology of extant mesembrinellids would suggest that *M*. *caenozoica* was also a stenotopic, silvicolous and shade-loving species. The extinction of mesembrinellids from the Caribbean islands is noteworthy, and *M*. *caenozoica* is another instance among dozens, where a widespread clade, documented from the Caribbean through a species preserved in Dominican amber, has experienced a local extinction (or nearly so) (topic most recently reviewed in Grimaldi *et al*. [55]). Most such extinctions are of insect groups that presently exist in Central or South America [56], as is the case for Mesembrinellidae. One explanation for these extinctions involves insularity and the geological history of the Caribbean plate: as the Proto-Caribbean land mass drifted away from nuclear America and became insular, the biota of the various islands that today form the Greater Antilles became depauperate. However, factors other than insularity could have driven some of the extinctions through time, in particular the changing palaeoenvironmental conditions that occurred through the Late Miocene. The mesembrinellids may also have become extinct on the Caribbean islands more recently as a consequence of the Plio-Pleistocene cooling, which resulted in extensive habitat disturbance and drying [57]. The narrow ecological requirements of the mesembrinellids make them particularly sensitive to climate change, which is a major determinant of habitat loss and fragmentation.

### Oestroid phylogeny: Faint light at the end of the tunnel?

Our phylogenetic analyses of Oestroidea are preliminary. The goal of our molecular analysis was not so much a focused effort to produce a robust phylogeny of the entire clade, but rather to employ available data to arrive at the best estimate of oestroid phylogeny, which served as a framework for roughly estimating ages of diversification for major lineages, based (in part) on this newly discovered oestroid fossil. In this light, we were surprised at how well these data recovered previously hypothesized clades (e.g., families, subfamilies) and how consistent many of the relationships were among different analyses (e.g., Mesembrinellidae + Ulurumyiidae; Rhinophoridae (Polleniinae + Tachinidae); Oestridae + Sarcophagidae). Still, higher ‘backbone’ relationships within the superfamily are not well resolved and differ among analyses, and a more intensive study employing many more loci will likely be needed to resolve them and produce a stable phylogenetic topology.

The estimated ages of key nodes in this study are largely consistent with other recent estimates of divergence times using a variety of molecular data sets, which have estimated the origin of Calyptratae at ca. 55 mya [1], 50.4 mya [11] and 60.4 mya [58]. Our estimate is similar, but somewhat older, at 67.5 my, indicating that calyptrates may date from before the K–Pg event. This similarity may stem partly from reliance on the same (few) fossil calibrations, although we are the first to include an undisputed oestroid fossil. Our results for the origin of Oestroidea (48.2 mya) and of the family Tachinidae (33.7 mya) are highly congruent with previous estimates (e.g., 56.0 and 33.2 mya [58], and ca. 40.0 and 30.0 mya [1]). A recent phylogenomic study of the insects as a whole [59] also suggests relatively recent phylogenetic origins for calyptrates and oestroids in the Upper Paleogene and Lower Oligocene, respectively. Remarkably, however, this study suggests that the split between Sarcophagidae and Tachinidae is approximately at the same depth as that between the apine genera *Apis* Linnaeus and *Bombus* Latreille (Hymenoptera: Apidae), if not younger. Regardless of the exact time of phylogenetic origin, the Calyptratae have likely experienced multiple episodes of rapid radiation, diversifying into about 22,000 described (and many undescribed) species in a relatively short time [1]. It may not be a coincidence that the origin of the calyptrates is estimated to be around the Cretaceous-Palaeogene mass extinction event. The upheaval of existing ecological communities and the opening of new niches following such a massive extinction event may have facilitated the diversification of lineages and life histories in this ecologically plastic clade of flies, as has been suggested for mammals, passerine birds, and other taxa [60, 61].

The mesembrinellid clade (including *Ulurumyia macalpinei*) appears to be of late Eocene age (43 myo), with the Mesembrinellidae estimated as about 39 myo, substantially older than the fossil of *M*. *caenozoica*. Given the uncertainty about where the fossil taxon fits among other *Mesembrinella* species in our molecular phylogeny, it is difficult to ascertain where the fossil calibration constraint should be placed on the phylogeny. It is possible that constraining a subgroup to a minimum age of 15 my, rather than the whole family, could push our estimated node ages further back. The near-basal position of Mesembrinellidae within the Oestroidea, isolated from other lineages of Calliphoridae *sensu lato*, was suggested previously by Crosskey [50] and Pape [62], although the close association with Ulurumyiidae is a novel hypothesis.

Morphology and molecules have usually yielded markedly different phylogenetic reconstructions of the oestroid lineages, and this applies to our analyses as well. Two exemplar cases involve the sister group relationship of the Tachinidae and the monophyly/non-monophyly of the bot flies. All recent phylogenetic reconstructions based on multiple genes [16, 17, 63] converge in reconstructing the calliphorid genus *Pollenia* as sister to a monophyletic Tachinidae with strong statistical support. Remarkably, there are very few clues supporting this hypothesis on morphological or ecological grounds, except that larvae of both *Pollenia* and ‘lower’ tachinids [36, 64] appear to be parasitoids of soil-dwelling organisms (i.e., earthworms and larvae of litter-associated weevils, respectively) [64]. On the other hand, molecular-based reconstructions often fail in retrieving Oestridae as monophyletic ([16, 17] in part), ([18] in part), [19], despite strong support from morphological data [12, 13, 28, 29] (Fig 3). As remarkable exceptions, all analyses presented here agree in reconstructing the family Oestridae as monophyletic and, for the first time, *Ulurumyia macalpinei* as sister to Mesembrinellidae, with this clade in turn being sister to the remaining Oestroidea (or at least much of it, Figs 3 and 5).

This situation exemplifies the uncertainty and difficulty in reconstructing relationships among oestroid lineages. Disagreement among reconstructions could be attributed to a possible rapid diversification event of persistent lineages [1, 17]. However, our time-calibrated phylogeny suggests that oestroid diversification as a whole may have been relatively ‘slow’ but punctuated by multiple episodes of more rapid radiation throughout the Cenozoic (Fig 5). On the other hand, the early evolution of oestroids could have been a time of ‘evolutionary experimentation’ during which many early lineages went extinct, leaving a somewhat skeletal phylogeny with only a few surviving lineages that later diversified. Without a better fossil record, it is impossible to discern between these possible scenarios.

Phylogenetic trees inferred from multiple gene sequences are starting to converge into similar, more stable patterns [59, 65, 66, 67, 68], and this is true also for the oestroids despite the sparse and uneven taxon coverage [16, 17]. If we assume that the recent molecular data are better at recovering the true phylogeny of Oestroidea, then we are still left with the question of why the morphology is so misleading and what this suggests about character evolution in this group. Conversely, if we assume inferences from the morphological data are closer to the truth, why are molecular-based inferences less accurate? Presently, we have a limited set of morphological characters with which to infer the phylogeny of the Oestroidea and this is due to the slow pace of detailed morphological investigation in recent decades. For many clades there are only one or two supporting character states, which are often homoplastic (S1 Fig). From this, it follows that very few additional characters, alternative homology assessments, or the use of different methods, might cause substantial topological changes to the inferred phylogenies. Also, there is no simple way to assess the complexity of morphological character evolution, except by their implied weights based on the number of homoplastic occurrences (but see [69]).

Differences between molecular- and morphology-based reconstructions of Oestroidea may seem unbridgeable, but the discovery of the first, undisputed oestroid fossil allows us to better establish the age and the ‘tempo and mode’ of diversification in these flies. Our results suggest that the K–Pg extinction event may have played a crucial role in boosting calyptrate diversification through the Cenozoic, as it did for the major radiations of birds, mammals and angiosperms.

## Materials and methods

### Amber inclusion

The amber inclusion was acquired from Mr. Jorge Martínez of Santiago, Dominican Republic, whose well-known workshop and business has provided Dominican amber inclusions for scientific study for decades. The mine source of the specimen is impossible to determine with certainty, since rough amber from various mines located north-northeast of Santiago [70] is usually mixed during processing in the workshops in Santiago. It is certain that the specimen is not copal (subfossilized resin that is only hundreds to thousands of years old, outcrops of which occur in eastern Dominican Republic in the vicinity of Cotui) because it is not reactive with organic solvents and is not the typical very light yellow colour of copal. The inclusion with the male holotype of *Mesembrinella caenozoica* sp. nov. is in the James Zigras collection, housed in the Division of Invertebrate Zoology at the American Museum of Natural History, New York.

### CT-scan analysis

Scanning was done in the Microscopy and Imaging Facility (MIF) at the American Museum of Natural History, using a General Electric Phoenix v|tome|x-s nanotube high resolution computed tomography system with a tungsten target, an accelerating voltage of 90kV and a current of 200μA, at resolutions between 9 and 7.5 μm. 300ms exposures were taken at 0.2° intervals. Volumetric data was produced with GE Phoenix’s Datos Reconstruction 2.2.1 software using a cone-beam filtered back projection reconstruction algorithm. A beam hardening correction was applied. Volumetric data was exported as16-bit integer greyscale TIFF stacks. Image stacks were combined in the Fiji distribution of NIH’s ImageJ with the Pairwise 3D stitching plug-in. Imaging focused on the male terminalia, in order to visualize hidden but informative features of the phallus such as the dorsolateral processes, and the cerci. Data were rendered and animated using Volume Graphics Studio Max 2.2.6 software equipped with the Coordinate Measurement Module.

### Morphological terminology

Morphological terminology follows Cumming & Wood [71], with minor differences as discussed in Cerretti *et al*. [35].

### Morphological cladistic analysis

To evaluate the phylogenetic position of *Mesembrinella caenozoica* sp. nov. among extant Oestroidea a matrix was constructed of 74 morphological characters for 49 taxa (S1 Dataset), mostly adapted from previous studies [12, 13, 14] with minor adjustments from our own studies. Oestroid diversity is represented by a sample of 45 species, including *M*. *caenozoica* described here, plus four muscoid outgroups (three Muscidae, one Anthomyiidae). The sample includes all oestroid subfamilies, except Paramacronychiinae (Sarcophagidae) (see S2 Text for a complete list of included taxa and depositories of material studied).

The data matrix was produced in Mesquite version 3.03 [72] (S1 Dataset). Cladistic analysis was conducted with TNT version 1.5 [73, 74]. Heuristic searches were run with the ‘traditional search’ option with the following parameters: General RAM of 1 GB, memory set to hold 1,000,000 trees, setting 1,000 replicates with tree bisection-reconnection (TBR) branch swapping and saving 1,000 trees per replicate. Multistate characters were treated as unordered and zero-length branches were collapsed. Inapplicable and unknown states were coded as ‘-’ and ‘?’, respectively, in Mesquite. Character state changes were optimized in WinClada version 1.00.08 [75]. The favoured tree was selected among the most parsimonious trees by calculating the total fit (command ‘fit*’ in TNT) for every tree under a range of k-values (S3 Text), using the unambiguous transformation algorithm, and choosing one of the trees with highest fit. Bremer support values were calculated in TNT from 15,000 trees up to 10 steps longer than the shortest trees obtained from a ‘traditional search’, using the ‘trees from RAM’ setting. Consistency and retention indices were calculated in TNT.

### Molecular phylogenetic analyses and divergence time estimation

Sequence data for 89 taxa was obtained either directly via PCR amplification of extracted DNA from collected specimens or through deposited sequence data from NCBI Genbank (see S2 Text). Most 28S and 16S sequences of mesembrinellids and some other oestroids were from Marinho *et al*. [15, 19], and most tachinid sequences, as well as a number of CAD sequences of other taxa, were from Winkler *et al*. [17]. For the newly obtained sequences, flies were collected by hand netting and 1–3 legs were removed from each specimen and placed in 95% ethanol shortly after collection. DNA extraction and isolation were performed with a Puregene® Tissue Kit (Qiagen Inc.) using standard methods (see [17]). PCR amplifications employed the primers 28SF and 28SR for the 5’ half of the 28S rDNA gene and 54F and 405R for the 5’ region of the CAD gene (‘CAD1’; see Winkler *et al*., [17], Moulton & Wiegmann [76]). PCR amplification protocols and conditions followed those outlined in Winkler *et al*. [17].

Sequences from each locus were aligned separately with MAFFT v.7 [77] via the online MAFFT server (http://mafft.cbrc.jp/alignment/server/), employing the G-INS-1 algorithm for CAD and the L-INS-I algorithm for 28S and 16S. A large unalignable intron of variable length in the centre of the fragment was trimmed from CAD [76]. Final sequence lengths were 1650 bp, 1615 bp, and 665 bp for 28S, CAD, and 16S respectively. In a few cases, concatenated sequences of different genes were from different congeneric species (see S2 Text). The full alignment is available through the online data repository TreeBASE (treebase.org).

A Maximum Likelihood analysis was performed using GARLI v2.01 [78]. The concatenated data set was partitioned by locus, and within CAD by codon position, and a separate GTR+I+Γ substitution model was defined for each partition, following the best partition and substitution model scheme given by PartitionFinder v1.1.1 [79], with all parameters estimated from the data. GARLI run was set as follows: 5 independent search replicates, 5x10^6^ generations and default options for automated stop. The hippoboscoid *Glossina morsitans* Westwood was selected as outgroup. 100 non-parametric bootstraps of the data set were conducted using the original search parameters, except for the genthreshfortopoterm option, which was set to 10,000. Similar results were obtained in a RaXML-HPC v.8.2.8 [80] run conducted on XSEDE servers via the CIPRES web portal (v. 3.3) [81], with the same parameters.

Bayesian phylogenetic inferences were performed with MrBayes 3.2.6 [82] and using BEAST v. 2.4.4 [83], both also conducted on XSEDE servers via the CIPRES web portal (v. 3.3) [81]. MrBayes analysis was conducted with the same partition and substitution model scheme used in the ML analysis and the run was set as follows: 50x10^6^ generations with two sets of 6 chains, sample frequency = 1,000 and burn-in set to 25% after checking for convergence. Node supports were assessed by analysing the *posterior* probabilities in the 50% extended majority-rule consensus tree. The BEAST analysis was set similarly to the MrBayes run and additional priors (for divergence time estimation) included a Yule coalescent model with parameters estimated with a gamma prior for relative birth rate, gamma shape parameters with exponential priors, and all rates with gamma distributed priors using default values of alpha and beta. The hippoboscoids (*Glossina morsitans* and *Ornithoctona erythrocephala* (Leach)) were assigned as the outgroup (i.e., all other taxa were constrained to be monophyletic). A relaxed log-normal clock model was used to estimate divergence times [84, 85], with an exponential distributed mean prior (ucldMean.c; mean = 10) and a gamma distributed standard deviation. Four calibrations were used at four nodes in the tree, in each case using a log normal distributed prior for the estimated tmrca (time to most recent common ancestor), and all were assumed to define a monophyletic group. These calibrations included: 1. Phaoniinae (Muscidae) from 15–20 myo Dominican amber [22], used to constrain *Phaonia* Robineau-Desvoidy + *Cordiluroides* Albuquerque (initial prior offset = 15); 2. *Glossina* Wiedemann (Glossinidae) from 35 myo Colorado shale [2], used to constrain *Glossina* + *Ornithoctona* Speiser (offset = 35); 3. *Protanthomyia* Michelsen (stem group Anthomyiidae) [20] from ca. 42 myo Baltic amber, used to constrain Anthomyiidae (offset = 42), and the current fossil *Mesembrinella* from 15–20 myo Dominican amber used to constrain the minimum age of Mesembrinellidae as a whole (offset = 15). Prior parameters for each of these distributions were estimated with log-normal hyper-prior distribution, beginning with M = 2.0, and S = 2.50. MCMC chain length was 50x10^6^ generations with trees stored every 1,000 generations and parameters logged every 1,000 generations. A conservative burn-in frequency of 25% was used based on visual examination of tree likelihood convergence using TRACER. TreeAnnotator v2.4.0 was used to calculate the maximum clade credibility tree and posterior probabilities of nodes based on the 37 500 trees retained by the MCMC analysis.

### Nomenclatural Acts

The electronic edition of this article conforms to the requirements of the amended International Code of Zoological Nomenclature, and hence the new names contained herein are available under that Code from the electronic edition of this article. This published work and the nomenclatural acts it contains have been registered in ZooBank, the online registration system for the ICZN. The ZooBank LSIDs (Life Science Identifiers) can be resolved and the associated information viewed through any standard web browser by appending the LSID to the prefix “http://zoobank.org/”. The LSID for this publication is: urn:lsid:zoobank.org:pub: D808DBAD-EE10-466F-94E5-A4B536EE938A. The electronic edition of this work was published in a journal with an ISSN, and has been archived and is available from the following digital repositories: PubMed Central, LOCKSS.

## Supporting information

S1 Text Detailed description of *Mesembrinella caenozoica* sp. nov. with morphological remarks. (DOCX)

S2 Text Schizophoran taxa included in the morphological and molecular phylogenetic analyses. (DOCX)

S3 Text Total fit calculated for every MPT tree under a range of k-values. (DOCX)

S1 Dataset Nexus file containing the dataset matrix for the phylogenetic analysis and MP trees. (TXT)

S1 Fig Favoured most parsimonious tree of Oestroidea from analysis of the morphological dataset, with character states traced. (PDF)

S2 Fig Maximum likelihood (ML) phylogenetic reconstruction of major lineages of Oestroidea from analysis of the combined (16S, 28S, CAD) dataset, generated in GARLI. ML bootstrap values are shown above branches.
(TIF)

S3 Fig Bayesian phylogenetic reconstruction of major lineages of Oestroidea from analysis of the combined (16S, 28S, CAD) dataset, generated in MrBayes. Posterior probability values are shown above branches.
(TIF)
