# Supplementary material for: First fossil of an oestroid fly (Diptera: Calyptratae: Oestroidea) and the dating of oestroid divergences
Source: PLoS One. 2017 Aug 23;12(8):e0182101. doi: 10.1371/journal.pone.0182101 (PMC5568141; doi:10.1371/journal.pone.0182101)
Supplement: S1 Text — (DOCX) [file pone.0182101.s001.docx]

**Supplementary Information (S1 text)**

**First fossil of an oestroid fly (Diptera: Calyptratae: Oestroidea) and the dating of oestroid divergences**

Pierfilippo Cerretti, John O. Stireman III, Thomas Pape, James E. O’Hara, Marco A. T. Marinho, Knut Rognes, David A. Grimaldi

urn:lsid:zoobank.org:pub:0DC5170B-1D16-407A-889E-56EED3FE3627

***Mesembrinella caenozoica* sp. nov.**

**Description**

Ground colour: [*It is not possible to precisely discern the ground colour of certain sclerites because there is a thin reflective layer of air, trapped by the microtomentum, evenly covering most of the body surface*.] Fronto-orbital plate, parafacial area, and gena yellowish-brown. Frontal vitta black. Scape and pedicel dark brown; postpedicel mostly dark brown except yellowish on proximo-ventral portion; arista yellow, only slightly darkened at base of third aristomere. Palpus dark brown. Thorax uniformly black or dark brown. Fringes of prothoracic and metathoracic spiracles dark brown. Tegula and basicosta dark. Wing membrane apparently hyaline, without darkened areas. Upper calypter whitish; lower calypter infuscated. Femora and tibiae appearing blackish-brown, or even somewhat reddish or yellowish when viewed from certain angles. Tarsi and claws uniformly dark brown. Pulvilli infuscate. Abdomen appearing uniformly black or dark brown, or even extensively red when viewed from certain angles.

Microtomentum: Fronto-orbital plate, parafacial area and gena evenly covered with thick microtomentum. Scutum densely microtomentose, with three well outlined longitudinal dark vittae running from presutural portion to postsutural portion. Abdomen appearing entirely covered with dense microtomentum.

Head (text Fig 1A,B): Head holoptic, distinctly higher than long. Compound eye bare. Eye facets enlarged on anteromedial area, border indistinct. Ocelli present, strongly approximated and ocellar triangle raised on small tubercle at vertex. Frons almost entirely obliterated from anterior ocellus to about midlength. Upper reclinate and proclinate orbital setae absent. Outer vertical seta fine and slightly differentiated from postocular setae. Inner vertical seta well developed (i.e., about same size as ocellar seta). Ocellar seta well developed, proclinate. One postocellar seta. Occiput with a well-defined row of fine postocular setae alternating in size. One row of 8 frontal setae descending to level of upper margin of scape. Parafacial and fronto-orbital plate bare. Parafacial at its narrowest point about 1/3 as wide as width of postpedicel. Parafacial strongly narrowing ventrad, at narrowest point about 1/3–2/5 its width at level of base of antenna. Facial ridge concave with decumbent, short setulae on lower 1/4–1/5. Vibrissa strong, arising at level of lower facial margin. Face [= facial plate, clypeus] slightly concave, non-carinate and not visible in lateral view. Lower facial margin warped forward and visible in lateral view in front of vibrissal triangle. Subvibrissal ridge moderately developed, with 3 setae. Genal dilation well developed, though very narrow. Gena very narrow, about 0.05 times as high as compound eye. Dorsal occiput strongly concave (setulation not visible). Ventral occiput covered with fine, black setulae only. Pedicel with several long setulae dorsally and one long, fine seta about half as long as arista. Postpedicel about 3.5 times as long as pedicel. Postpedicel narrow in lateral view, gently concave along anterodorsal margin (i.e., from arista to apex), and slanted posteroapically. Arista long plumose (i.e., total width of arista and microtrichia more than twice the width of postpedicel), thickened only on proximal 1/5–1/6. First and second aristomeres short, not longer than wide. Prementum about 0.65–0.70 times as long as height of head. Labella broad and about 4/5 as long as prementum. Palpus sub-cylindrical, about as long as antenna, with setae irregular in length and thickness along its length.

Thorax (text Fig 1C): Prosternum and prosternal membrane bare. Proepisternal depression with pale, hair-like setulae. Proepisternal seta present and directed upwards, about half as long as proepimeral seta. Prothoracic spiracle large, elliptical, with a single lappet, opening dorsally. Postpronotum with 3 setae arranged in a triangle. Scutum with 3(presutural) [4 on right side, but anterior one short and weak] + 3(postsutural) acrostichal setae, 3(presutural) + 3(postsutural) dorsocentral setae (anterior presutural dorsocentral seta doubled on both sides), 1(presutural) + 2(postsutural) intra-alar setae (postsutural intra-alar setae divided by a distance greater than the distance between anterior seta and transverse suture), 1(presutural) + 3(postsutural) supra-alar setae (first postsutural supra-alar seta slightly longer than presutural intra-alar seta). Three posthumeral setae; outer posthumeral seta standing laterad to a line through the supra-alar setae. Two notopleural setae, posterior one shorter. Postalar callus with two strong setae, an additional weak, third seta present posteriorly. Postalar wall with a few setulae. Three katepisternal setae (2+1). Coxopleural streak absent. Katepimeron with a few hair-like setulae anteriorly. Anepimeron with a patch of short fine setae; i.e., anepimeral seta not differentiated. Lower part of anterior half of anepimeron bare. Metathoracic spiracle large, with a single, reniform lappet with dorsal opening [Mesembrinellidae groundplan]. Metathoracic spiracular lappet without setae. Meral setae arranged in a vertical row in front of and below metathoracic spiracle. Metakatepimeron bare. Katatergite bare (i.e., non-setose). Anatergite not visible. Scutellum with 3 pairs of marginal setae (basals, laterals, and apicals), and one pair of discal setae. Apical marginal scutellar setae crossed, sub-horizontal, about as long as basal setae; lateral setae fine, about 4/5 as long as apicals. Ventrolateral sides of scutellum with scattered, short, black setulae. Subscutellum well developed, moderately convex; dorsal half not sclerotized. General setulae of postpronotum, scutum and scutellum relatively long and suberect. Lower calypter rounded, posteriorly broad. Posterolateral margin of lower calypter with long trichia.

Wing: Tegula with row of setae; basicosta bare. Stem vein bare. Subcostal sclerite bare. Costal spine not differentiated from costal setae. [Underside of costal vein not visible.] Subcostal vein with a distinct concavity. Vein R_4+5_ with fine setulae from base to about 2/3 of distance to crossvein r-m. Section of vein M between crossveins r-m and dm-m distinctly longer than section between dm-m and bend of M. Bend of M broadly rounded, postangular section of M very close to and parallel with wing margin. Vein CuA+CuP not reaching wing margin. Alula well developed and narrow.

Legs: Fore tibia with indistinct anterodorsal setae, 1 posterior seta and with anterodorsal preapical seta distinctly shorter than dorsal preapical seta. Fore claws and pulvilli longer than fifth tarsomere. Mid tibia with 1 anterodorsal seta and 1 submedian ventral seta. Hind tibia with 2 anterodorsal setae, 1 posterodorsal seta and 2 dorsal preapical setae (anterodorsal and dorsal). Posterior margin of hind coxa appears to be bare [area not clearly visible].

Abdomen: Broadly oval. Mid-dorsal depression of syntergite 1+2 reaching posterior margin of syntergite. Syntergite 1+2 without median marginal setae and with two short, weak lateral marginal setae. Tergite 3 with two long, erect median marginal setae and two lateral marginal setae. Tergites 4 and 5 with a row of erect marginal setae. Discal setae absent. General setulae of abdomen prone to sub-erect (especially ventrally and mid-dorsally). Abdominal sternites exposed; setae on sternites normally developed and not arranged in two rows.

Terminalia (text Fig 2): Posterior margin of sternite 5 with a deep median notch; lateral lobe rounded posteriorly, basal desclerotized ‘window’ not visible. Tergite 6 broad, not indented posteriorly and not fused to syntergosternite 7+8 (text Fig 2A) (a continuous deep groove separates these two sclerites, suggesting they are likely connected by membrane). [Sternite 6 not visible.] Syntergosternite 7+8 broad. Epandrium short, convex, with a well-developed lateral lobe. Cerci (posterior view) not fused medially, distally pointed and sub-parallel (Fig 2B). Cercus (lateral view) long, narrow and evenly curved anteroventrally (text Fig 2C,D), basally thick, narrowing distally and sharply pointed at tip. Surstylus long and narrow, evenly curved inward with distal half slightly flattened laterally (posterior view; text Fig 2B); straight in lateral view and apically rounded (text Fig 2D). [Although it is not possible to distinguish between sclerites and membranes, the complete basal silhouette of the surstylus cannot be traced and this suggests that the surstylus is at least partly fused to the epandrium as in all extant mesembrinellids.] Bacilliform sclerite discernible as a shallow ridge, attached (or articulated) to anterobasal corner of surstylus (Fig 2E). Pregonite short, hook-shaped; postgonite long, narrow and evenly curved anteriorly (Fig 2D,E). Epiphallus attached dorsobasally to basiphallus, well-developed, long, evenly bent ventrally and narrowing distally. Extension of dorsal sclerite of distiphallus separated longitudinally; tip of extension of dorsal sclerite of distiphallus separate from distiphallus wall (Fig 2D–E). Median process of ventral sclerite of distiphallus not visible. Lateroventral lobes of distiphallus well developed; small sclerotized spines of lateroventral lobes of distiphallus not visible [possibly due to insufficient resolution of CT scan]; narrow lateral projection of lateroventral lobes of distiphallus present. Acrophallus well developed, sub-cylindrical (Fig 2C,D).

**Remarks**

The CT scan of the male terminalia of *M. caenozoica* revealed (i) a well-developed and complete suture between tergite 6 and syntergosternite 7+8 (text Fig 2A), (ii) long, narrow and evenly curved cerci (text Fig 2B), and (iii) the presence of narrow, lateroventral projections of distiphallus (text Fig 2C,D). These three character states of the terminalia are crucial to understand the phylogenetic affinities of this Dominican species among mesembrinellids. In particular, the complete suture between tergite 6 and syntergosternite 7+8 (text Fig 1A), despite being plesiomorphic, excludes placement of *M. caenozoica* within clade D (text Fig 3) because the fused condition is an autapomorphy of the latter. The shape of the male cerci is a nonhomoplastic apomorphy supporting monophyly of clade E. Moreover, the presence of narrow lateroventral projections of the distiphallus supports placement of *M. caenozoica* as sister to *M. facialis* (text Fig 3). The latter species is readily distinguishable from *M. patriciae*, *M. perisi*, *M. nigripes* and *M. caenozoica* by being robust, more than 12 mm in length, and by having broad and subrectangular abdominal sternites, each provided with two rows of robust submarginal setae. Unfortunately, it is not possible to determine whether *M. caenozoica* possesses the other autapomorphy of *M*. *facialis*, i.e., the sclerotized membrane between tergite 6 and syntergosternite 7+8. Nevertheless, tergite 6 and syntergosternite 7+8 in *M. caenozoica* are very close together as in *M. facialis*, whereas in *M. perisi* and *M. nigripes* they are clearly separated. *Mesembrinella patriciae* is ambiguous for this trait (i.e., coded with ‘?’) as the original paper is contradictory. In fact, Wolff (2013) describes tergite 6 and syntergosternite 7+8 as ‘partly fused’ in *M. patriciae*, but the ink drawing (Wolff, 2013: figure 2B) shows a complete suture between the two sclerites.

*Mesembrinella caenozoica* sp. nov. seems devoid of any metallic colour or reflections, which is a feature shared with *M. perisi*, *M. nigripes* and *M. facialis* [according to Wolff (2013), *M. patriciae* has a metallic sheen on abdominal tergites]. However, as colour and reflectivity are rarely preserved in amber inclusions (DG, personal observations), separate analyses were run coding *M. caenozoica* as either ‘unknown’ or ‘non-metallic’ for this trait. No differences were observed in tree topologies nor in branch supporting values. Metallic reflections seem to have evolved several times independently across the Calyptratae and in particular in the Oestroidea (Cerretti, Pape, unpubl.). In our analysis the metallic sheen of the cuticle (character 1, state 1), in the mesembrinellids, has been reconstructed as independently evolved in the ancestor of clade D and in *M. patriciae*.

*Mesembrinella caenozoica* sp. nov. is characterized by one unique and likely derived character state among Mesembrinellidae, namely the presence of a row of fine setulae on the dorsal side of wing vein R_4+5_ that extends from the base to about 2/3 of the distance to crossvein r-m.

**References**

Wolff, M. A new species of *Mesembrinella* (Diptera: Calliphoridae: Mesembrinellinae) from Colombia. Revista Colombiana de Entomología 2013; 39: 120–124.
