# Supplementary material for: First fossil of an oestroid fly (Diptera: Calyptratae: Oestroidea) and the dating of oestroid divergences
Source: PLoS One. 2017 Aug 23;12(8):e0182101. doi: 10.1371/journal.pone.0182101 (PMC5568141; doi:10.1371/journal.pone.0182101)
Supplement: S2 Text — (DOCX) [file pone.0182101.s002.docx]

**Supplementary Information (S2 text)**

**First fossil of an oestroid fly (Diptera: Calyptratae: Oestroidea) and the dating of oestroid divergences**

Pierfilippo Cerretti, John O. Stireman III, Thomas Pape, James E. O’Hara, Marco A. T. Marinho, Knut Rognes, David A. Grimaldi

urn:lsid:zoobank.org:pub:0DC5170B-1D16-407A-889E-56EED3FE3627

**S2 Text.** Schizophoran taxa included in the morphological and molecular phylogenetic analyses. Most mesembrinellid sequences are from Marinho *et al*. (2012, 2016) and most tachinid sequences are from Winkler *et al*. (2015). Abbreviations: A = adult; L1 = first instar; L3 = third instar. When sequences for different loci were from different congeneric species, they are separated by a “/”. Acronyms of repositories, in square brackets: AMNH = American Museum of Natural History, NY, USA; MZUR = Museo di Zoologia, Sapienza Universitá di Roma, Roma, Italy; NMB = National Museum, Bloemfontein, South Africa; ZMUC = Natural History Museum of Denmark, University of Copenhagen, Copenhagen, Denmark; JOSC = John O. Stireman, private collection.

| Family | Subfamily | Species | Source: morphology / molecules [Collection acronym]^1^ | Morphology^2^, stages examined | 16S | 28S | CAD | [stage: reference] / GenBank Accessions (16S/28S/CAD) |
| --- | --- | --- | --- | --- | --- | --- | --- | --- |
| Glossinidae | -- | *Glossina morsitans* Westwood | GenBank |  | x | x |  | JQ246760/JQ246656 |
| Hippoboscidae | Ornithomyiinae | *Ornithoctona erythrocephala* (Leach) | GenBank |  |  | x |  | JQ246761/JQ246657 |
| Fanniidae | -- | *Fannia canicularis* (L.) | GenBank |  | x | x | x | DQ648647/ AJ867935 /FJ025514 |
| Muscidae | Azeliinae | *Muscina stabulans* (Fallén) | GenBank |  |  | x | x | EF531145/ AJ867938 |
| Muscidae | Coenosiinae | *Cordiluroides megalopyga* Albuquerque | GenBank |  |  |  | x | AJ867933 |
| Muscidae | Muscinae | *Musca autumnalis* De Geer | Italy [MZUR] | A, L1, L3 |  |  |  |  |
| Muscidae | Muscinae | *Musca domestica* L. | GenBank |  | x | x | x | JQ246756/AJ551427/ AY280689 |
| Muscidae | Phaoniinae | *Phaonia subventa* (Harris) / *Phaonia shannoni* Carvalho & Pont | GenBank |  |  | x | x | KJ476360/ AJ867943 |
| Muscidae | Reinwardtiinae | *Alluaudinella centralis* Malloch | Kenya [MZUR] | A, L1, L3 |  |  |  | [L3: Skidmore, 1985] |
| Muscidae | Reinwardtiinae | *Ochromusca trifaria* (Bigot) | South Africa [MZUR] | A, L1 |  |  |  | [L3: Skidmore, 1985] |
| Anthomyiidae | Anthomyiinae | *Anthomyia* sp. | Italy [MZUR] | A, L1, L3 |  |  |  |  |
| Anthomyiidae | Anthomyiinae | *Botanophila fugax* (Meigen) | GenBank |  |  | x |  | DQ656967 |
| Anthomyiidae | Anthomyiinae | *Eustalomya* sp. | GenBank |  |  |  | x | AJ867946 |
| Anthomyiidae | Anthomyiinae | *Hylemya vagans* (Panzer) / *Hylemya* sp. | GenBank |  |  | x | x | FJ025528/AJ605059 |
| Scathophagidae | Scathophaginae | *Scathophaga tinctinervis* (Becker) | GenBank |  |  | x |  | DQ657032 |
| Calliphoridae | Ameniinae | *Amenia* sp. | Australia (NSW) [MZUR] | A, L1 |  |  |  | [A, L1, L3: Crosskey, 1965; Colless, 1998] |
| Calliphoridae | Ameniinae | *Catapicephala* spp. | -- | -- |  |  |  | [A, L1, L3: Crosskey, 1966; Colless, 1998] |
| Calliphoridae | Aphyssurinae | *Aphyssura* spp. | -- | -- |  |  |  | [A, L1: Norris, 1999] |
| Calliphoridae | Bengaliinae | *Auchmeromyia bequaerti* (Roubaud) | GenBank |  |  | x | - | JQ246626 |
| Calliphoridae | Bengaliinae | *Auchmeromyia* *senegalensis* (Macquart) | Kenya [MZUR] | A |  |  |  | L1, L3: Rognes, 1997; Pape & Arnaud, 2001 |
| Calliphoridae | Bengaliinae | *Bengalia* spp. | Kenya [MZUR] | A |  |  |  |  |
| Calliphoridae | Bengaliinae | *Bengalia* *peuhi* Villeneuve / *Bengalia* sp. | Vietnam [JOSC] |  | x | x | x | JQ246734**/** KY945985**/** KY928446 |
| Calliphoridae | Bengaliinae | *Cordylobia anthropophaga* (Blanchard) | GenBank |  | x | x |  | JQ246730/AJ551432 |
| Calliphoridae | Bengaliinae | *Hemigymnochaeta unicolor* (Bigot) | GenBank |  | x | x |  | JQ246731/JQ246628 |
| Calliphoridae | Calliphorinae | *Calliphora vicina* Robineau-Desvoidy | GenBank |  | x | x |  | JQ246721/JQ246617 |
| Calliphoridae | Calliphorinae | *Calliphora vomitoria* (L.) | Italy [MZUR] / GenBank | A, L1, L3 | x | x |  | JQ246722/AJ300133 |
| Calliphoridae | Calliphorinae | *Onesia tibialis* (Macquart) | GenBank |  |  | x |  | AJ558188 |
| Calliphoridae | Calliphorinae | *Protocalliphora azurea* (Fallén) / *Protocalliphora sialia* Shannon & Dobroscky | GenBank |  | x | x |  | GQ409147/ AJ558190 |
| Calliphoridae | Chrysomyinae | *Chrysomya megacephala* (Fabricius) | GenBank |  | x | x |  | KT272775/AJ551435 |
| Calliphoridae | Chrysomyinae | *Chrysomya* spp. | Italy / Kenya / South Africa | A, L1, L3 |  |  |  |  |
| Calliphoridae | Chrysomyinae | *Cochliomyia hominivorax* (Coquerel) | GenBank |  | x | x |  | JQ246714/JQ246610 |
| Calliphoridae | Chrysomyinae | *Phormia regina* (Meigen) | GenBank |  | x | x |  | JQ246718/JQ246614 |
| Calliphoridae | Helicoboscinae | *Eurychaeta muscaria* (Meigen) | Italy [MZUR] | A, L1, L3 |  |  |  |  |
| Calliphoridae | Luciliinae | *Lucilia cuprina* (Wiedemann) | GenBank |  | x | x |  | JQ246726/FR719302 |
| Calliphoridae | Luciliinae | *Lucilia sericata* (Meigen) | GenBank |  |  | x | x | JQ246728/KP954369/KP973901 |
| Calliphoridae | Luciliinae | *Lucilia* spp. | Italy / Greece / South Africa | A, L1, L3 |  |  |  |  |
| Calliphoridae | Melanomyinae | *Melanomya bicolor* (Coquillett) / *Melanomya abdominalis* (Reinhard) | U.S.A. [JOSC] |  |  | x | x | KY945988 / KP973909 |
| Calliphoridae | Melanomyinae | *Melinda* sp. | Italy [MZUR] | A |  |  |  |  |
| Calliphoridae | Parameniini | *Paramenia* spp. | Australia (NSW) [MZUR] | A |  |  |  | [A, L1, L3: Crosskey, 1966; Rognes, 1997; Colless, 1998] |
| Calliphoridae | Phumosiinae | *Euphumosia* sp. | -- | -- |  |  |  |  |
| Calliphoridae | Phumosiinae | *Phumosia* sp. | South Africa [MZUR] | A, L1 |  |  |  |  |
| Calliphoridae | Polleniinae | *Morinia doronici* (Scopoli) | Italy [MZUR] | A |  |  |  |  |
| Calliphoridae | Polleniinae | *Pollenia pediculata* (Macquart) | GenBank |  |  | x | x | KP954375/ KP973925 |
| Calliphoridae | Polleniinae | *Pollenia rudis* (Fabricius) | Italy [MZUR] / GenBank | A, L1, L3 | x | x |  | KR820849/ GQ409263 |
| Calliphoridae | Polleniinae | *Morinia* sp. | South Africa [MZUR, NMB, ZMUC] | A |  |  |  |  |
| Calliphoridae | Toxotarsinae | *Sarconesia chlorogaster* (Wiedemann) | GenBank |  |  | x |  | JQ246723/JQ246619 |
| Calliphoridae | Toxotarsinae | *Sarconesia* sp. | Chile [MZUR] | A, L1 |  |  |  |  |
| Calliphoridae | ?Toxotarsinae | *Toxotarsus ambrosiana* (Lopes) | Chile [AMNH] | A |  |  |  |  |
| Mesembrinellidae | -- | *Mesembrinella nigripes* (Guimarães) | -- / GenBank | -- | x | x |  | [A, L1, L3: Guimarães, 1977] / KR820851/ KR820888 |
| Mesembrinellidae | -- | *Mesembrinella perisi* (Mariluis) | -- / GenBank | -- | x | x |  | [A: Wolff et al., 2013] / KR820850/ KR820887 |
| Mesembrinellidae | -- | *Mesembrinella caenozoica* **sp. nov.** | Dominican amber inclusion [AMNH] | A (holotype male only) |  |  |  |  |
| Mesembrinellidae | -- | *Mesembrinella latifrons* (Mello) | -- | -- |  |  |  | [A, Guimarães, 1977] |
| Mesembrinellidae | -- | *Mesembrinella cyaneicincta* (Surcouf) | GenBank |  | x | x |  | KR820873/ KR820914 |
| Mesembrinellidae | -- | *Mesembrinella quadrilineata* (Fabricius) | GenBank |  | x | x |  | JQ246736/ JQ246633 |
| Mesembrinellidae | -- | *Mesembrinella randa* (Walker) | GenBank |  | x | x |  | KR820876/ KR820920 |
| Mesembrinellidae | -- | *Mesembrinella carvalhoi* (Wolff *et al*.) | GenBank |  | x | x |  | KR820880/ KR820921 |
| Mesembrinellidae | -- | *Mesembrinella aeneiventris* (Wiedemann) | GenBank |  |  | x |  | KR820922 |
| Mesembrinellidae | -- | *Mesembrinella vogelsangi* (Mello) | GenBank |  |  |  |  | KR820926 |
| Mesembrinellidae | -- | *Mesembrinella* sp. 1 (*obscura*) | GenBank |  | x | x |  | KR820882/ KR820923 |
| Mesembrinellidae | -- | *Mesembrinella* sp. | Ecuador [JOSC] |  |  | x | x | KP954372/ KP973922 |
| Mesembrinellidae | -- | *Mesembrinella apollinaris* Séguy | GenBank |  | x | x |  | KR820853/ KR820891 |
| Mesembrinellidae | -- | *Mesembrinella batesi* Aldrich | GenBank |  | x | x |  | KR820854/ KR820894 |
| Mesembrinellidae | -- | *Mesembrinella bellardiana* Aldrich | GenBank |  | x | x |  | KR820859/ KR820897 |
| Mesembrinellidae | -- | *Mesembrinella bicolor* (Fabricius) 1 | GenBank |  | x | x |  | JQ246740/ /JQ246637 |
| Mesembrinellidae | -- | *Mesembrinella bicolor* (Fabricius) 3 | GenBank |  | x | x |  | JQ246741/ JQ246638 |
| Mesembrinellidae | -- | *Mesembrinella currani* Guimarães | GenBank |  | x | x |  | KR820863/ KR820901 |
| Mesembrinellidae | -- | *Mesembrinella patriciae* Wolff | GenBank | A | x | x |  | [A: Wolff, 2013, Marinhho et al. 2017] / KR820864/ KR820902/ |
| Mesembrinellidae | -- | *Mesembrinella peregrina* Aldrich | GenBank |  | x | x |  | KR820866/ KR820903 |
| Mesembrinellidae | -- | *Mesembrinella pictipennis* Aldrich | GenBank |  | x | x |  | KR820865/ KR820904 |
| Mesembrinellidae | -- | *Mesembrinella townsendi* Guimarães | GenBank |  | x | x |  | KR820869/ KR820908 |
| Mesembrinellidae | -- | *Mesembrinella* sp.1 | Costa Rica [MZUR] | A, L1 |  |  |  |  |
| Mesembrinellidae | -- | *Mesembrinella* sp.2 | Guyana [MZUR] | A, L1 |  |  |  |  |
| Mystacinobiidae | -- | *Mystacinobia zelandica* Holloway | New Zealand [ZMUC, MZUR] / GenBank | A | x | x |  | [A, L1, L3: Rognes, 1997; Pape & Arnaud 2001]/GQ409136/ JF439567 |
| Oestridae | Cuterebrinae | *Cuterebra* sp./ *Cuterebra austeni* Sabrosky | GenBank |  | x | x | x | JQ246753/KP954361/KP973914 |
| Oestridae | Cuterebrinae | *Cuterebra* sp. | U.S.A [MZUR] | A |  |  |  | [L1, L3: Pape, 2001; Sabrosky, 1986] |
| Oestridae | Gasterophilinae | *Gasterophilus intestinalis* (De Geer) | Italy [MZUR] | A, L1, L3 |  |  |  |  |
| Oestridae | Hypodermatinae | *Hypoderma lineatum* (Villers) | Italy [MZUR] | A, L1, L3 |  |  |  |  |
| Oestridae | Oestrinae | *Cephenemyia jellisoni* Townsend | GenBank |  |  | x | x | KP954359/ KP973912 |
| Oestridae | Oestrinae | *Oestrus ovis* L. | Italy [MZUR] | A, L1, L3 |  |  |  |  |
| Rhiniidae | -- | *Isomyia gomezmenori* (Peris) | GenBank |  |  | x |  | JF439579 |
| Rhiniidae | -- | *Rhyncomya impavida* (Rossi) | Italy [MZUR] | A |  |  |  |  |
| Rhiniidae | -- | *Rhinia* sp. | GenBank |  | x | x |  | JQ246743/JQ246640 |
| Rhiniidae | -- | *Rhyncomya* sp. | South Africa [JOSC] |  |  | x | x | KY945989**/** KY928447 |
| Rhiniidae | -- | *Rhyncomya soyauxi* Karsch/ *Rhyncomya nigripes* (Séguy) | GenBank |  |  | x |  | JQ246744/ GQ409268 |
| Rhinophoridae | -- | *Axinia lucaris* Colless | Australia [ZMUC] | A |  |  |  |  |
| Rhinophoridae | -- | *Bezzimyia yepezi* Pape & Arnaud | Costa Rica [ZMUC] | A, L1 |  |  |  | [L1: Pape & Arnaud, 2001] |
| Rhinophoridae | -- | *Bezzimyia* sp. | Ecuador [JOSC] |  |  |  | x | KY928451 |
| Rhinophoridae | -- | *Oplisa* sp. (cf. *tergestina* (Schiner)) | Italy [JOSC] |  |  | x | x | KY945987**/** KY928448 |
| Rhinophoridae | -- | *Phyto discrepans* Pandellé | Italy [MZUR] | A |  |  |  | [L1, L3: Bedding, 1973] |
| Rhinophoridae | -- | *Rhinomorinia sarcophagina* (Schiner) | Slovenia [JOSC] |  |  | x | x | KY945990**/** KY928449 |
| Rhinophoridae | -- | *Rhinophora lepida* (Meigen) | Italy [MZUR] | A, L1 |  |  |  | [L1, L3: Bedding, 1973] |
| Rhinophoridae | -- | *Stevenia* *hertingi* Kugler / *Stevenia* sp. | Italy [JOSC] |  |  | x | x | GQ409162**/**KY945991**/** KY928450 |
| Sarcophagidae | Miltogramminae | *Macronychia* sp. | GenBank |  |  | x | x | KP954370/KP973921 |
| Sarcophagidae | Miltogramminae | *Metopia campestris* (Fallén) | GenBank |  |  | x |  | JF439573 |
| Sarcophagidae | Miltogramminae | *Miltogramma* sp. | Thailand | A, L1 |  |  |  |  |
| Sarcophagidae | Sarcophaginae | *Helicobia rapax* (Walker) | GenBank |  |  | x | x | KP954366/ KP973918 |
| Sarcophagidae | Sarcophaginae | *Peckia ingens* (Walker) | GenBank |  | x | x |  | JQ246747/JQ246643 |
| Sarcophagidae | Sarcophaginae | *Sarcophaga carnaria* (L.) | GenBank |  |  | x |  | AJ551430 |
| Sarcophagidae | Sarcophaginae | *Sarcophaga shirakii* (Kano & Field) | GenBank |  |  | x |  | AB466110 |
| Sarcophagidae | Sarcophaginae | *Sarcophaga* sp. | Italy [MZUR] | A, L1, L3 |  |  |  |  |
| Sarcophagidae | Sarcophaginae | *Tripanurga* sp. | GenBank |  |  | x |  | AF366678 |
| Tachinidae | Dexiinae | *Campylocheta semiothisae* (Brooks) | GenBank |  |  | x | x | KP954357 / KP973900 |
| Tachinidae | Dexiinae | *Dexia rustica* (Fabricius) | Italy [MZUR] | A, L1, L3 |  |  |  |  |
| Tachinidae | Dexiinae | *Epigrimyia illinoensis* Robertson | GenBank |  |  | x | x | KP954364/ KP973915 |
| Tachinidae | Dexiinae | *Euthera setifacies* Brooks | GenBank |  |  | x | x | KP954365/ KP973916 |
| Tachinidae | Dexiinae | *Ptilodexia conjuncta* (Wulp) | GenBank |  |  | x | x | KP954376/ KP973897 |
| Tachinidae | Dexiinae | *Thelaira americana* Brooks | GenBank |  |  | x | x | KP954381/ KP973929 |
| Tachinidae | Dexiinae | *Uramya* sp. | GenBank |  |  | x | x | KP954383/ KP973931 |
| Tachinidae | Dexiinae | *Voria ruralis* (Fallén) | GenBank |  |  | x | x | KP954384/ KP973898 |
| Tachinidae | Exoristinae | *Blondelia hyphantriae* (Tothill) | GenBank |  |  | x | x | KP954356/ KP973910 |
| Tachinidae | Exoristinae | *Exorista larvarum* (L.) | Italy [MZUR] | A, L1, L3 |  |  |  |  |
| Tachinidae | Exoristinae | *Gonia ornata* Meigen | Italy [MZUR] | A, L1, L3 |  |  |  |  |
| Tachinidae | Exoristinae | *Hyphantrophaga hyphantriae* (Townsend) | GenBank |  |  | x | x | KP954367 KP973919 |
| Tachinidae | Exoristinae | *Lespesia aletiae* (Riley) | GenBank |  |  | x | x | KP954368/ KP973920 |
| Tachinidae | Exoristinae | *Tachinomyia nigricans* Webber | GenBank |  |  | x | x | KP954380/ KP973896 |
| Tachinidae | Exoristinae | *Winthemia sinuata* Reinhard | GenBank |  |  | x | x | KP954385/ KP973932 |
| Tachinidae | Phasiinae | *Catharosia* sp. cf. *nebulosa* (Coquilett) | GenBank |  |  | x | x | KP954358/ KP973911 |
| Tachinidae | Phasiinae | *Cylindromyia binotata* (Bigot) | GenBank |  |  | x | x | KP954362/ KP973906 |
| Tachinidae | Phasiinae | *Ectophasia crassipennis* (Fabricius) | Italy [MZUR] | A |  |  |  | [L1: Dupuis, 1963] |
| Tachinidae | Phasiinae | *Gymnosoma nudifrons* Herting/ *Gymnosoma nitens* Meigen/ *Gymnosoma par* Walker | GenBank |  | x | x | x | GQ409130/ GQ409239/ KP973917 |
| Tachinidae | Phasiinae | *Phasia* sp. | GenBank |  |  | x | x | KP954374/ KP973924 |
| Tachinidae | Phasiinae | *Strongygaster triangulifera* (Loew) | GenBank |  |  | x | x | KP954379/ KP973928 |
| Tachinidae | Phasiinae | *Trichopoda pennipes* (Fabricius) | GenBank |  |  | x | x | KP954382/ KP973930 |
| Tachinidae | Tachininae | *Ceracia dentata* (Coquillett) | GenBank |  |  | x | x | KP954360/ KP973913 |
| Tachinidae | Tachininae | *Epalpus signifer* (Walker) | GenBank |  |  | x | x | KP954363/ KP973899 |
| Tachinidae | Tachininae | *Gnadochaeta* sp. | U.S.A [MZUR] | A, L1 |  |  |  |  |
| Tachinidae | Tachininae | *Panzeria ampelus* (Walker) | GenBank |  |  | x | x | KP954373/ KP973923 |
| Tachinidae | Tachininae | *Siphona plusiae* Coquillett | GenBank |  |  | x | x | KP954378/ KP973927 |
| Tachinidae | Tachininae | *Tachina fera* (L.) | Italy [MZUR] | A, L1, L3 |  |  |  |  |
| Ulurumyiidae | -- | *Ulurumyia macalpinei* Michelsen & Pape (McAlpine’s fly) | Australia [MZUR] | A, L1 | x | x | x | [L1, L3: Ferrar, 1979] /Q409133/ KY945986 **/** KY928452 |

^1^The locality is given only for the examined material, otherwise it refers to the molecular data depository.

^2^In the case of morphological data retrieved only from the literature (abbreviation: “--”), see the reference section of main text.

**References**

Bedding RA. The immature stages of Rhinophorinae (Diptera: Calliphoridae) that parasitise British woodlice. Transactions of the Royal Entomological Society of London 1973; 125: 27–44.

Colless DH. Morphometrics in the genus *Amenia* and revisionary notes on the Australian Ameniinae (Diptera: Calliphoridae), with the description of eight new species. Records of the Australian Museum 1998; 50: 85–123.

Crosskey RW. A systematic revision of the Ameniinae (Diptera: Calliphoridae). Bulletin of the British Museum (Natural History). Entomology 1965; 16: 33–140.

Dupuis C. Essai monographique sur les Phasiinae (Diptères Tachinaires parasites d’Hétéroptères). Mémoires du Muséum National d’Histoire Naturelle. Series A (Zoologie) 1963; 26, 1–461.

Ferrar P. The immature stages of the dung-breeding muscoid flies in Australia,

with notes on the species, and keys to larvae and puparia. Australian Journal of

Zoology, Supplement 1979; 73: 1–106.

Guimarães JH. A systematic revision of the Mesembrinellidae, stat. nov. (Diptera, Cyclorrhapha). Arquivos de Zoologia 1977; 29: 1–109. doi: 10.11606/issn.2176-7793.v29i1p1-109

Marinho MAT, Wolff M, Ramos-Pastranab Y, Lima de Azeredo-Espind AM, Amorim DdeS. The first phylogenetic study of Mesembrinellidae (Diptera: Oestroidea) based on molecular data: clades and congruence with morphological characters. Cladistics 2017; 33: 134–152. doi: 10.1111/cla.12157

Norris KR. Establishment of a subfamily Aphyssurinae for the Australian genus *Aphyssura* Hardy (Diptera: Calliphoridae), with a review of known forms and descriptions of new species. Invertebrate Taxon 1999; 13: 511-628.

Pape T. Phylogeny of Oestridae (Insecta: Diptera). Systematic Entomology 2001; 26: 133–171. doi: 10.1046/j.1365-3113.2001.00143.x

Pape T, Arnaud PH Jr. *Bezzimyia* – a genus of native New World Rhinophoridae (Insecta, Diptera). Zoologica Scripta 2001; 30: 257–297. doi: 10.1046/j.1463-6409.2001.00064.x

Rognes K. The Calliphoridae (blowflies) (Diptera: Oestroidea) are not a monophyletic group. Cladistics 1997; 13: 27–66. doi: 10.1006/clad.1997.0031

Wolff M. A new species of *Mesembrinella* (Diptera: Calliphoridae: Mesembrinellinae) from Colombia. Revista Colombiana de Entomología 2013; 39: 120–124.

Wolff M, Ramos-Pastrana Y, Pujol-Luz JR Description of the male of *Laneella perisi* (Mariluis) (Diptera: Calliphoridae) n. comb. Neotropical Entomology 2013; 42:58-62. doi: 10.1007/s13744-012-0092-2
