# Supplementary material for: First fossil of an oestroid fly (Diptera: Calyptratae: Oestroidea) and the dating of oestroid divergences
Source: PLoS One. 2017 Aug 23;12(8):e0182101. doi: 10.1371/journal.pone.0182101 (PMC5568141; doi:10.1371/journal.pone.0182101)
Supplement: S3 Text — (DOCX) [file pone.0182101.s003.docx]

**Supplementary Information (S3 text)**

**First fossil of an oestroid fly (Diptera: Calyptratae: Oestroidea) and the dating of oestroid divergences**

Pierfilippo Cerretti, John O. Stireman III, Thomas Pape, James E. O’Hara, Marco A. T. Marinho, Knut Rognes, David A. Grimaldi

urn:lsid:zoobank.org:pub:0DC5170B-1D16-407A-889E-56EED3FE3627

**S3 Text.** Total fit calculated for every tree under a range of k-values. Fittest trees in bold followed by an asterisk; favoured tree, chosen for character optimization (S1 Fig), underlined.

K = 3 [TNT command ‘piwe=3; fit*’]

49.06 – 49.12 – 49.08 – 49.09 – 49.06 – 49.12 – 49.11 – 49.08 – 49.09 – 49.08 – 49.06 – 49.06 – **49.13*** – 49.12 – 49.11 – 49.08 – 49.08 – 49.09 – 49.09 – 49.08 – 49.12 – 49.11 – 49.08 – 49.08 – 49.06 – 49.09 – 49.10 – 49.10 – **49.13*** – **49.13***

K = 4 [TNT command ‘piwe=4; fit*’]

51.97 – 52.02 – 51.98 – 51.99 – 51.97 – 52.02 – 52.01 – 51.98 – 51.99 – 51.98 – 51.97 – 51.97 – 52.02 – 52.02 – 52.01 – 51.98 – 51.98 – 51.99 – 51.99 – 51.98 – 52.01 – 52.01 – 51.98 – 51.98 – 51.98 – 51.99 – 52.01 – 52.01 – **52.03*** – **52.03***

K = 5 [TNT command ‘piwe=5; fit*’]

54.16 – 54.20 – 54.17 – 54.18 – 54.16 – 54.20 – 54.19 – 54.17 – 54.18 – 54.17 – 54.16 – 54.16 – **54.21*** – 54.20 – 54.19 – 54.17 – 54.17 – 54.18 – 54.18 – 54.17 – 54.20 – 54.19 – 54.17 – 54.17 – 54.17 – 54.18 – 54.20 – 54.20 – **54.21*** – **54.21***

K = 6 [TNT command ‘piwe=6; fit*’]

55.87 – 55.91 – 55.89 – 55.89 – 55.87 – 55.91 – 55.90 – 55.89 – 55.89 – 55.88 – 55.87 – 55.87 – 55.92 – 55.91 – 55.90 – 55.89 – 55.89 – 55.89 – 55.89 – 55.88 – 55.91 – 55.90 – 55.88 – 55.88 – 55.89 – 55.90 – 55.92 – 55.92 – **55.93*** – **55.93***

K = 7 [TNT command ‘piwe=7; fit*’]

57.27 – 57.30 – 57.28 – 57.28 – 57.27 – 57.30 – 57.29 – 57.28 – 57.28 – 57.27 – 57.27 – 57.27 – 57.30 – 57.30 – 57.29 – 57.28 – 57.28 – 57.28 – 57.28 – 57.27 – 57.30 – 57.29 – 57.27 – 57.27 – 57.28 – 57.29 – 57.31 – 57.31 – **57.32*** – **57.32***
