# Supplementary material for: First fossil of an oestroid fly (Diptera: Calyptratae: Oestroidea) and the dating of oestroid divergences
Source: PLoS One. 2017 Aug 23;12(8):e0182101. doi: 10.1371/journal.pone.0182101 (PMC5568141; doi:10.1371/journal.pone.0182101)
Supplement: S1 Fig — (PDF) [file pone.0182101.s005.pdf]

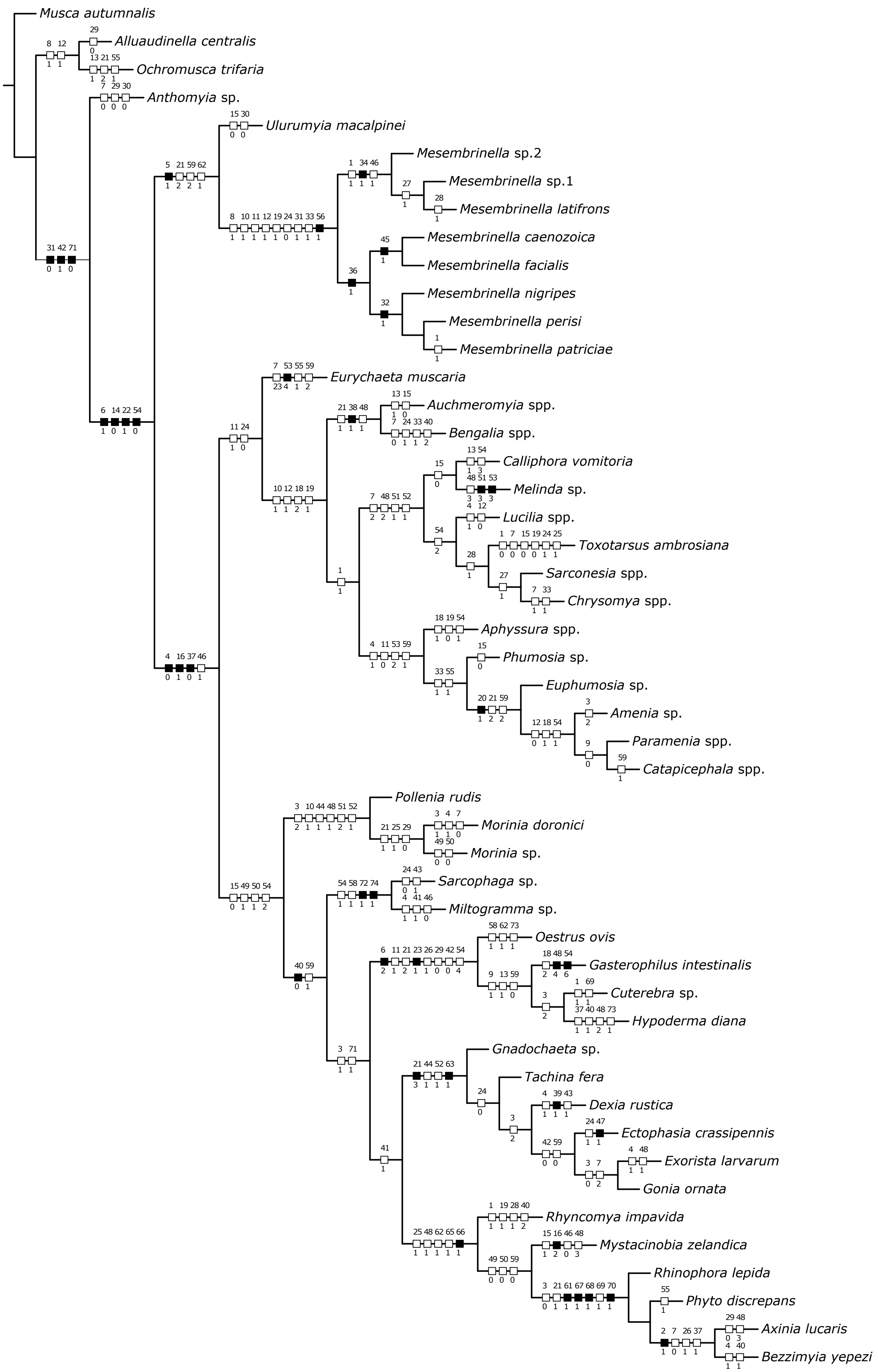

S1 Fig. Favoured most parsimonious tree of Oestroidea from analysis of the morphological dataset, with character states traced
